# Understanding Syrian migration in Lebanon: a methodological framework

**DOI:** 10.12688/openreseurope.16261.1

**Published:** 2023-09-06

**Authors:** Maria Gabriella Trovato, Nayla Al-Akl, Dana Ali

**Affiliations:** 1 Department of Landscape Architecture, Faculty of Landscape and Society, Norwegian University of Life Sciences, Ås, Norway; 2 Landscape Design and Ecosystem Management, American University of Beirut, Beirut, Beirut Governorate, Lebanon; 3 Independent researcher, Beirut, Lebanon

**Keywords:** Lebanon, Syrian displaced, Legal status and protection, ADMIGOV, Bekaa Valley, Saida, Development projects

## Abstract

**Background:** Wars, crises, and climate change are just a few of the worldwide concerns that have resulted in the forced relocation of millions of people. After 12 years of conflict in Syria, millions of Syrians are still displaced in the neighbouring countries, and their conditions have worsened due to the economic and socio-political crisis of the region. This paper reports on a study conducted in Lebanon as part of the EU Horizon-funded project ADMIGOV – Advancing Alternative Migration Governance. It describes the methodological framework used to study Syrian migration in Lebanon and sheds light on the phenomenon's patterns, challenges, and impacts.

**Methods:** In our study, we opted for a mixed method. It is built on a large corpus of primary data collected over the course of years of intensive, in-depth fieldwork and the author's immersion in the community. Alongside observations, quantitative and qualitative phone interviews were conducted to obtain the perceptions of displaced Syrians living in informal tented settlements in rural Lebanon and an incomplete building in the city of Saida. This interview data is accompanied by primary and secondary data sources, including the findings of other European research projects, statistics from UNHCR and IOM, and academic and press articles.

**Results:** Our research revealed the difficulties Syrians displaced in Lebanon encounter while navigating the challenging situation they are trapped in. The weakness of public institutions and their failure to guarantee the observance of basic human rights has compromised displaced safety. Moreover, even though the development interventions and aid assistance have been necessary for Syrians' survival, they proved inadequate and insufficient, evidencing the inefficiencies of most projects.

**Conclusions:** The findings contribute to an enriched understanding of the situation of Syrians in Lebanon and offer insights for policymakers, practitioners, and researchers working in the field of forced migration and humanitarian responses.

## Plain Language summary

Since the war began in 2011, more than 6.8 million Syrians have been displaced inside their own country, and another 5.5 million fled to neighbouring countries for safety ( UNHCR, 2023a). This study examines the forces contributing to the protracted displacement of Syrians in Lebanon and limiting the agency of displaced individuals. It explores the relationship between Syrian migration trends, socio–economic and political constraints and development interventions in the country to shed light on how these factors have shaped displaced vulnerability and marginalisation and influenced the deciding factors behind the migration or return decision.

Moreover, the research looks at the plight of Syrian refugees in Lebanon, emphasising how they are spatially marginalised and segregated from urban, peri-urban, and rural areas. Therefore, it investigates how Syrian refugees manage to make ends meet in the cramped conditions of their transitory homes on the peripheries of several urban clusters. Our research was informed by mapping activities, a literature review, and quantitative and qualitative interviews. The Bekaa Valley, with the horizontal expansion of Informal Tented Settlements (ITSs) on agricultural fields and the coast, specifically the city of Saida, were chosen as cases study. The research is part of the EU Horizon-funded project titled ADMIGOV – Advancing Alternative Migration Governance.

## Introduction

Since the war began in 2011, more than 6.8 million Syrians have been displaced inside their own country, and another 5.5 million fled to neighbouring countries for safety ( UNHCR, 2023a). The conflict has profoundly impacted Lebanon, which due to its geographic proximity, initial open borders strategy and longstanding bonds, became a significant refugee destination for those escaping Syria. Unfortunately, this migratory flow has severely tested Lebanon's precarious social, economic, and political system. About 1.5 million Syrians and a sizable group of Palestinian refugees make Lebanon the country with the world's biggest displaced population per capita ( UN, Government of Lebanon, 2019). More than 1,700 towns around the country are now hosting refugees, many of whom are also among the poorest in the country.

However, the Lebanese government has been reluctant to establish additional refugee camps to the already present Palestinian camps. As a result, 70% of Syrians have fled to large cities like Beirut, Saida or Tripoli, renting flats with family and friends. Others, 21%, settled in informal tented settlements in frontier areas, such as the northern Wadi Khaled and the eastern Bekaa Valley, and ten per cent live in non-residential shelters ( Kabbanji & Kabbanji, 2018; LCRP, 2023). Female-headed households are more likely than Syrian male-headed households to reside in informal settlements (27% vs 19%), with the majority concentrated in the Bekaa and Baalbek-Hermel governorates. Overall, 58% of displaced Syrians live in inadequate shelter conditions distributed across the three shelter types, with the highest percentage of inadequacy (78%) being in informal settlements ( Vasyr, 2022).

A mutual and precarious catastrophe for both Syrians and Lebanese people emerges from the presence of those vulnerable displaced populations in a heavily indebted middle-income nation like Lebanon. Since 2015, most of the new emigration has occurred in areas with high exposure to natural and human-caused risks, high socioeconomic vulnerability, and inadequate coping capability ( IDMC, 2017). Moreover, people's constant flow and mobility determine new spatial dynamics and social geographies ( Barnett, 2013), with intricate and far-reaching effects on Lebanon's society, economics, security, and politics. Understanding those repercussions is crucial for designing policies and actions that effectively address the needs of displaced Syrians and their host communities. In some Lebanese districts, Syrians have altered the ethnic and sectarian composition, contributing to increased social tensions and racial phenomena. In others, their presence has also affected access to basic services and strained local infrastructures. Disagreements on how to respond to the crisis from the political sphere have hampered the development and execution of effective solutions.

Lebanon is uniquely positioned as the country faces multiple crises, including socio-political, economic, and infrastructural challenges. Its financial and economic crisis has been ranked by the World Bank (2021) among the top 10 most severe episodes globally since the mid-nineteenth century. As the economy continues to decline, displaced Syrians and impoverished Lebanese rely more on aid assistance to make ends meet. Since late 2019, the Lebanese Lira has lost almost 100% of its value, and poverty rates have risen remarkably. ESCWA (2020) found that whereas 55% of the Lebanese lived below the poverty line, 90% of displaced Syrians were in extreme poverty. As conditions in Lebanon deteriorate, displaced Syrians are now stuck in a state of ‘limbo’, with a dignified return to a safe Syria not guaranteed, and resettlement to a third host country is a lengthy process granted to a few ( RPW, 2020, p. 5).

This study examines the systemic forces contributing to the protracted displacement of Syrians in Lebanon and limiting the agency of displaced individuals. It explores the relationship between Syrian migration trends, socio–economic and political constraints and development interventions in the country to shed light on how these factors have shaped displaced vulnerability and marginalisation and influenced the deciding factors behind the migration or return decision.

Moreover, the research looks at the plight of Syrian refugees in Lebanon, emphasising how they are spatially marginalised and segregated from urban, peri-urban, and rural areas. Therefore, it investigates how Syrian refugees manage to make ends meet in the cramped conditions of their transitory homes on the peripheries of several urban clusters. Many mapping activities, a literature review, and quantitative and qualitative interviews informed our research. The Bekaa Valley, with the horizontal expansion of Informal Tented Settlements (ITSs) on agricultural fields and the coast, specifically the city of Saida, were chosen as cases study.

### Syrians in Lebanon

The Syrian conflict has been a major cause of international migration in recent decades ( UNHCR, 2019). Since 2011, Syrians have fled to neighbouring countries like Turkey, Lebanon, Jordan, and Egypt. Lebanon hosts the world's largest number of refugees per capita, with the number of registered Syrian refugees by UNHCR reaching over a million in 2014 ( UNHCR, 2020). This was when Syrians, who were initially viewed as guests, became a threat that needed to be contained ( Uzelac & Meester, 2018). Indeed, Lebanon, which has not joined either the 1951 Refugee Convention or its 1967 Protocol pertaining to the Status of Refugees, has frequently stated that it is not a country of asylum but rather one of transit ( Fakhoury, 2017; Lebanon Support, 2020). This attitude demonstrates the uncertain governance of displaced Syrians with the rejection of formal camps, the termination of refugee registration by UNHCR in 2015, and the designation of refugees as displaced persons, and more recently, as "temporarily displaced persons" rather than refugees ( LCRP, 2019). The latter is understood as a response to a concern that the label "refugees" would make international refugee law applicable and provide the impression of permanence, as it did with Palestinian refugees ( Janmyr & Mourad, 2018).

But if we look back at history, the border between Lebanon and Syria has always been permeable, disputed, and ill-defined, with constant trespassing of the limits between the two countries ( Picard, 2006; Picard, 2016). Since the 1950s, many Syrians have worked in Lebanon, mostly in construction, public works, and agriculture ( Chalcraft, 2008). Some writers report the presence of 300,000 and 400,000 Syrians in Lebanon in 2011 ( Longuenesse, 2015), mostly males who migrated seasonally from Syria, leaving their families behind ( Kabbanji & Kabbanji, 2018). The economies of the border areas between the two nations were based on anything from massive official commerce to small-scale smuggling. At the beginning of 2011, this significant inflow of Syrians in Lebanon and the country’s exposure to varied degrees of armed combat altered the dynamics of these bordering areas. For the first three years of the war, the Lebanese government paid little attention to the plight of Syrian refugees, relying instead on the international community to shoulder the burden ( Trovato, 2021). This period is named the ‘open door policy’ and lasted until 2014. During this phase, UNHCR was the primary body overseeing refugee admission, reception, and registration. In May 2015, the Policy Paper on Syrian Displacement issued in 2014 was implemented, starting a period of a ‘locked door policy’, which is still in force today ( Fawaz *et al.,* 2018; Trovato *et al.,* 2021). As a result, UNHCR ceased registering Syrians as refugees, and the highly controversial sponsorship system for foreign workers became the primary legal entrance mechanism. Therefore, Syrians entering Lebanon after 2015 have been “recorded” rather than “registered” and are referred to as “temporarily displaced persons” ( Fawaz *et al.,* 2018; Janmyr & Mourad, 2018). In addition, this new policy imposed strict rules on short-term visas and long-term residency requirements that made it difficult for Syrians to enter or stay in Lebanon ( Lebanon Support, 2020). Besides, to minimise the number of Syrians in the country, in 2018 and 2019, the Ministry of Labour (MoL) launched a series of repressive actions targeting the informal labour market that exacerbated displaced living conditions. As a result, in 2020, 80% of Syrian refugees had no proper documentation for living in the country ( VASyR, 2019; VASyR, 2020). Additionally, GSO’s policies allowed specific actions of temporary arrest, arbitrary detention, and deportation ( CLDH, 2016). This increased the incidence of illegal immigration, stay and work, boosting clientelistic networks and practices of bribery and corruption ( Lebanon Support, 2015; Mencutek, 2017). So, in 2022, based on the Lebanon Crisis Response Plan ( LCRP, 2023), legal residency rates have remained low, with only 17% of displaced Syrians and 49% of Palestinian refugees from Syria attaining legal residency. Of the 1.500.000 million displaced Syrians, 83% are without legal residency, 64% without birth registration, and 66% without marriage registration. The lack of access to civil status documentation, including birth, marriage, divorce, and death, has implications on legal protection, including guardianship and inheritance rights.

Since 2014, when the state tightened its policy on asylum and residency, the lack of clarity and coherence in providing legal status for refugees has left Syrians unaware of their eligibility for humanitarian aid and their status ( Human Rights Watch, 2016: 21–23). According to Janmyr's research into the hierarchy of protection for different types of Syrian refugees in Lebanon, this lack of clarity creates an "additional layer of uncertainty for already-precarious displaced Syrians" ( Janmyr, 2018: 412). In fact, this illegitimate status affects every aspect of their life, including the ability to move freely without being stopped at checkpoints, to find work, to be paid the agreed-upon wage for their labour, and their safety from eviction. The lack of appropriate documents also makes local and international donors and organisations increasingly difficult to reach Syrians in need and to help them safely migrate or resettle in a third country. Additionally, the institutional ambiguity around the refugee response allows the state to expel refugees and evades responsibility ( Baumann & Kanafani, 2020). From 2011 to 2020, 2016 had the most significant number of resettlement departures, with 62,000 Syrians successfully relocated by UNHCR. In 2017, a repatriation program to return displaced safely and voluntarily was initiated by the Lebanese General Security Directorate (GSD), in collaboration with the Syrian intelligence services ( Dagher, 2021). Yet, as scholars argue, safety, voluntariness, and sustainability conditions are not fulfilled ( Içduygua & Nimer, 2020). Furthermore, as Lebanon's economic condition worsens, more and more people are looking for a way out, regardless of the consequences. Between January 2020 and May 2021, nearly 1,162 persons tried to exit Lebanon aboard smugglers' boats ( Sewell & Tamo, 2021), with only two out of eight boats making it to their destination due to Lebanese or Cypriot government interception or rejection. On the other hand, Lebanese authorities issued several acts of eviction and deportation. By April 2021, 433,000 people had returned to Syria since 2017, including Syrians, registered and non-registered as refugees, returning either on their own or with the help of the GSD (ibid.).

## Methods

### Methodological approach

In recent years, Lebanon has faced many crises resulting in a serious collapse. The economic crisis, political upheaval, the 2020 Beirut port disaster, and the COVID-19 epidemic have all contributed to structural vulnerability and a rise in poverty rates. The uniqueness of the country's circumstances since the October 2019 ‘uprising’ has influenced our methodological choice and the interview procedure. Therefore, we opted for a mixed method to understand the displaced community's experience, need, and perception of the situation they are trapped in. The method has been decided upon by the research work packages leader and partners and adapted to the specific country's situation. It is grounded in a large corpus of primary data gathered by in-depth, multi-year fieldwork and familiarity with the areas gained through years of presence in the selected locations. This field research includes monitoring locations and displaced Syrians' activities and conditions. Alongside observations, quantitative and qualitative phone interviews were conducted to hear from displaced Syrians living in informal tented settlements in rural Lebanon and an incomplete building in the city of Saida. The quantitative data collection aimed at producing systematic and structured data to describe the population under study's response to the development intervention and describe their overall condition and quality of life. The quantitative data have been matched and related to the qualitative info on participants’ experiences of access to aid and development projects and to legal, medical, and shelter protection.

The interviews are accompanied by secondary data sources, including academic and press articles, the findings of other European research projects, government records and publications, and data portals and repositories operated by international and local organisations concerned with migration statistics from UNHCR and IOM. Through in-depth content analysis, we mapped the state of the art on Syrians displaced in the region. Data from several sources have been triangulated to evaluate the reliability of the data provider, the data collection strategy, and any possible biases or constraints that might have influenced the validity of the data.

Two case studies have been profiled: Bar Elias and Saadnayel in Zahle, the main city of the Bekaa Governate, and Saida, a coastal city south of Beirut. The predominant type of accommodation in Zahle, Bekaa, is Informal Tented Settlements (ITS), while in Saida, the displaced inhabit residential and non-residential shelters and some ITS. The sites were chosen based on the researcher's previous knowledge and acquaintance with the location, the availability of data from previous years of study and the ease of access to the villages owing to ground contact. In Saida, we first focused on Syrian refugees at a collective shelter (the Ouzai shelter). However, the displaced who had been staying there were forced out in October of 2020, and our contacts resettled in several residential and residential buildings in and around the city.

### Ethical considerations

This study received ethical approval from the American University of Beirut's Institutional Review Board (IRB) under approval number SBS-2020-0132, which granted permission to conduct the study in Lebanon and requested to delete sensitive terminology and any detailed experiences that could have affected the respondents. Due to the COVID-19 restrictions, the IRB approved that the interviews be conducted by phone. The interlocutors orally consented to participate and publish the participant’s details after listening to the study’s goals and having the informant's letter read by the interviewers. Moreover, the coordinating partner of the project, the University of Amsterdam‘s standard procedures within its Amsterdam Institute of Social Science Research (AISSR) tested the ADMIGOV project on its ethical standards, and the ethics committee provided a guarantee and special expertise in dealing with ethical issues that may arise in the context of research.

The study involved collecting sensitive personal data such as ethnic background, migration route, and possibly health situation and political views of migrants, which can create problems for the interviewed community. The ADMIGOV consortium, aware of its high responsibility to deal with the ethical issues, developed strategies not to harm participants while protecting their safety, rights and interests, values above all and that human dignity is respected. Participation in the study was voluntary and targeted people over 18. The AUB team comprehended four female researchers that worked as data collectors and analysts in the project: Dana Ali, Sara Abou Faker, Rana Itani and Yasmin Abdul Nasser El Hakim. They possessed a master’s degree and were recruited based on their experience in conducting similar type of study. Researchers and interviewers were trained based on pre-field work instructions developed by the work package leader in contact with the ethics officers to ensure some coaching for the researchers. Pollers conducted the interviews from the American University of Beirut premises. When AUB was closed due to the pandemic, they conducted the work from their home in a sealed room. The confidentiality of the information provided by participants and their anonymity were of the utmost importance. All interviews were conducted anonymously, with no identifiers collected. Identifying information, such as specific names or locations, was anonymised on transcription/coding with names separated from data. There were no monetary incentives. Sensitive issues like political preferences and health status were avoided. Data were uploaded to the surfdrive provided by the University of Amsterdam and removed from our laptop. The recordings were deleted after transcription.

### Quantitative survey methodology

The quantitative questionnaire comprehended 10 sections. It included a background overview of the participants' personal history, such as their place of birth, level of education, the number of people and children in the household (HH), the number of working members in the HH, and if they had children, whether they had attended school in the previous month. Their present job situation, legal status, housing tenure status, primary income sources, home appliances ownership, and internet connectivity at home or by mobile phone were also examined. In addition, information on migration aspirations, development intervention policies, and risk attitudes was covered. The migration history, including routes, modes of transportation, and travel experiences, was also documented and encompassed migratory preferences, such as the desire to remain, leave for a third nation, or return to their home country, and the means and motivations for pursuing onward migration. Since the research was conducted during the COVID-19 pandemic, we also recorded the influence of the epidemy on migration intentions, employment, and money and the perceptions of the COVID-19 threat on a global, national, and family level. The questions on development interventions informed whether, in the previous 12 months, the displaced had received assistance or remittances and development aid such as training or counselling services. Moreover, participants were asked to rate their living conditions, healthcare access, education, employment opportunities, future ambitions, and ties to the local community.

For the quantitative survey, 185 interviews of 40 to 45 minutes were conducted between February and July 2021 in Saida, Bar Elias and Saadnayel. The sampling was selected with the help of the Dutch Refugee Council (DRC) operating in Lebanon, which supplied an initial set of respondents, ‘seeds’ per the study locations. This initial cohort started the ball rolling by recruiting others in their social circles to take part in the surveys we conducted. All participants provided informed verbal consent before the phone interviews after the pollers read an informant letter with the study’s goals. Every effort was made to ensure that there was a roughly equal number of male and female participants across all case study locations. In Saida, where the study's intended participants had previously lived in a shared shelter, and participant recruiting was simpler over the phone, the gender balance was easy to reach, while it was more difficult in Bekaa where we recruited 42 men and 25 females. The interviews were conducted by phone due to the COVID-19 restriction in accessing the study locations, and audio recordings were made. The interviewees seemed eager to share their perspectives and personal experiences. The questionnaire was developed by the study work package partners and tailored to the country's characteristics and the population we surveyed. It complies with the ethical guidelines of Amsterdam University, the lead partner of the project. The survey was also examined by the Institutional Review Board (IRB) of the American University of Beirut, which approved conducting the study in Lebanon and asked to remove sensitive terminology and any detailed experiences that could have affected the respondents. All interviews were done in Arabic, the participants' preferred language, and adhered to the guidelines drafted by the University of Amsterdam. Throughout the surveys, the interviewers took notes and typed the responses into an Arabic template. Then, they used the recordings to fill in any blanks or shed light on any confusing topics. Alongside these steps, the pollers reviewed, cleaned up and translated all the responses into English. All data were examined for any participant identity (and subsequently wiped) in accordance with the ADMIGOV Data Management strategy and ethical processes, and data were also stored on encrypted computers accessible only by the researchers.

### Qualitative method

The qualitative interviews included closed and open-ended questions and were conducted in two separate phases. In the first round (October 2020– January 2021), 24 displaced Syrians were equally divided amongst the two case studies, Zahle's Bar Elias and Saadnayel, as well as Saida's Ouzai shelter (before eviction), and between male and female, when possible. The survey questionnaire features 61 questions corresponding to seven sections. Protection in asylum-seeking, settling, employment, medical care, entering and leaving the country, and challenges and needs, were covered. In this phase, participants were recruited with the help of the Dutch Refugee Council (DRC) in Lebanon. DRC supplies the contact information for focal points (FP) in the research locations. FP provided us with the phone number of some displaced Syrians residing in our case studies. Through snowball sampling, we recruited 24 Syrians displaced over 18 living in the two Informal settlements in Zahle and in the unfinished building in Saida. In the second round (April – May 2021), we interviewed 30 participants, with a balance between males and females. The interviews, which lasted around 25 minutes, comprised 29 open-ended questions revolving around development interventions, plans to stay/migrate, factors in the decision to stay/migrate, migration decision-making processes, and displaced future plans. In this phase, participants were a sampling of the 185 polled for the quantitative interviews. They were the 30 living in the two Informal settlements in Zahle and in the unfinished building in Saida that agreed to be questioned in a second round. Survey participation was voluntary and anonymous, and interviewees were eager to share their experiences with us. Due to the COVID-19 pandemic, access to the venues was restricted, so the interviews took over the phone. All respondents verbally agreed to participate in the research after being informed about the project's aims and having an information letter read by phone before the interview.

The questionnaires comply with the ethical guidelines of Amsterdam University, the lead partner of the project. They were also reviewed by the American University of Beirut's Institutional Review Board (IRB), which granted the ethical permission to conduct the study in Lebanon and requested to delete sensitive terminology and any detailed experiences that could have affected the respondents. All interviews followed the interview guidelines drafted by the University of Amsterdam. They were done in Arabic, the participants' preferred language.

During the surveys, notes were taken, and responses were typed into an Arabic template; recordings of the interviews were then used to fill in any gaps or clarify any points that were unclear. A review, clean-up, and English translation of the interviews followed. In accordance with the ADMIGOV Data Management strategy and ethical processes, all data were carefully reviewed for any participant identification (and then erased), and data was also stored on encrypted computers accessible only to the researchers.

### Data analysis

The quantitative and qualitative data from the interviews were analysed using two different software tools. SPSS.25 software (Statistical Package for the Social Sciences version 25) was used to analyse the quantitative data, and categorical variables were reported numerically. GNU, a freely accessible PSPP software, is also capable of the same analysis used in this study. QRS NVivo 12 was used as a software for interpreting and analysing the qualitative data. Taguette, a freely accessible software, is also capable of the same analysis used in this study. The interviews were recorded, transcribed, anonymised, translated, and coded in two stages: manually and using the software. Data were coded with labels related to the key topics of the research, allowing the emerged patterns to be identified in categories. Patterns were then examined and placed together to let overarching themes emerge ( Percy *et al*., 2015). Any additional second-level pattern that arose was categorised as a secondary theme. Following, the data were interpreted using a mixed inductive and deductive strategy to determine the correctness of the hypotheses and if the emerging themes were in line with the research contents. Researchers adopted an inductive (or ‘Grounded’) methodology to learn more about participants' lived experiences in coping with difficult situations. Coding and thematic analysis of the data revealed underlying key themes and trends. However, given the amount of prior studies and analyses, a deductive approach was also employed. The analysis was conducted in accordance with the existing body of knowledge, as well as the guidelines and research aim outlined in the project’s mandate.

### Secondary sources

Several sources were used to compile information and set up a structure for determining where gaps in the understanding of protection and development existed in the literature. Sources were identified based on the main topics of our project: protection and development intervention in governing the refugee crisis. Scientific articles shed light on the latest research within the migration field and particularly on the condition of Syrian refugees in the region. Fakhoury, Janmyr, and Romola are some of the scholars we mostly relied on for the extensive work they have done in the past on Syrian displaced conditions in Lebanon. Moreover, they provided info on important factual background related to the governance strategy and the process of the legal status of the displaced. Data and official statistics were retrieved from official government papers and 'grey literature' from the inter- and non-governmental sectors. Among these are the UNHCR and UNOCHA reports, as well as the UNHCR, UNICEF, and WFP joint report titled "VasYr" ( 2019; 2020). Reports from the European Commission, Food and Agriculture Organisation, and Lebanon Crisis Report plan have also been used to retrieve statistics on development interventions and response strategies. Our investigation into protection, including examining legislative laws, migration governance, and refugee protection regimes, was grounded in the Lebanon Support reports ( 2016; 2018; and 2020) as part of the Horizon 2020 RESPOND project on migration and protection. To learn more about how COVID-19 and Lebanon's economic crisis have affected Syrian refugees, we looked at the work of many NGOs, such as the DRC, Norwegian Refugee Council (NRC), Human Rights Watch (HRW), Solidarités International (SI), and SEED. Updates on the present situation of Syrian refugees were also found in online media stories from outlets including the Daily Star, Aljazeera News, and the Middle East Institute, among others.

### Case studies method

**Figure 1.**
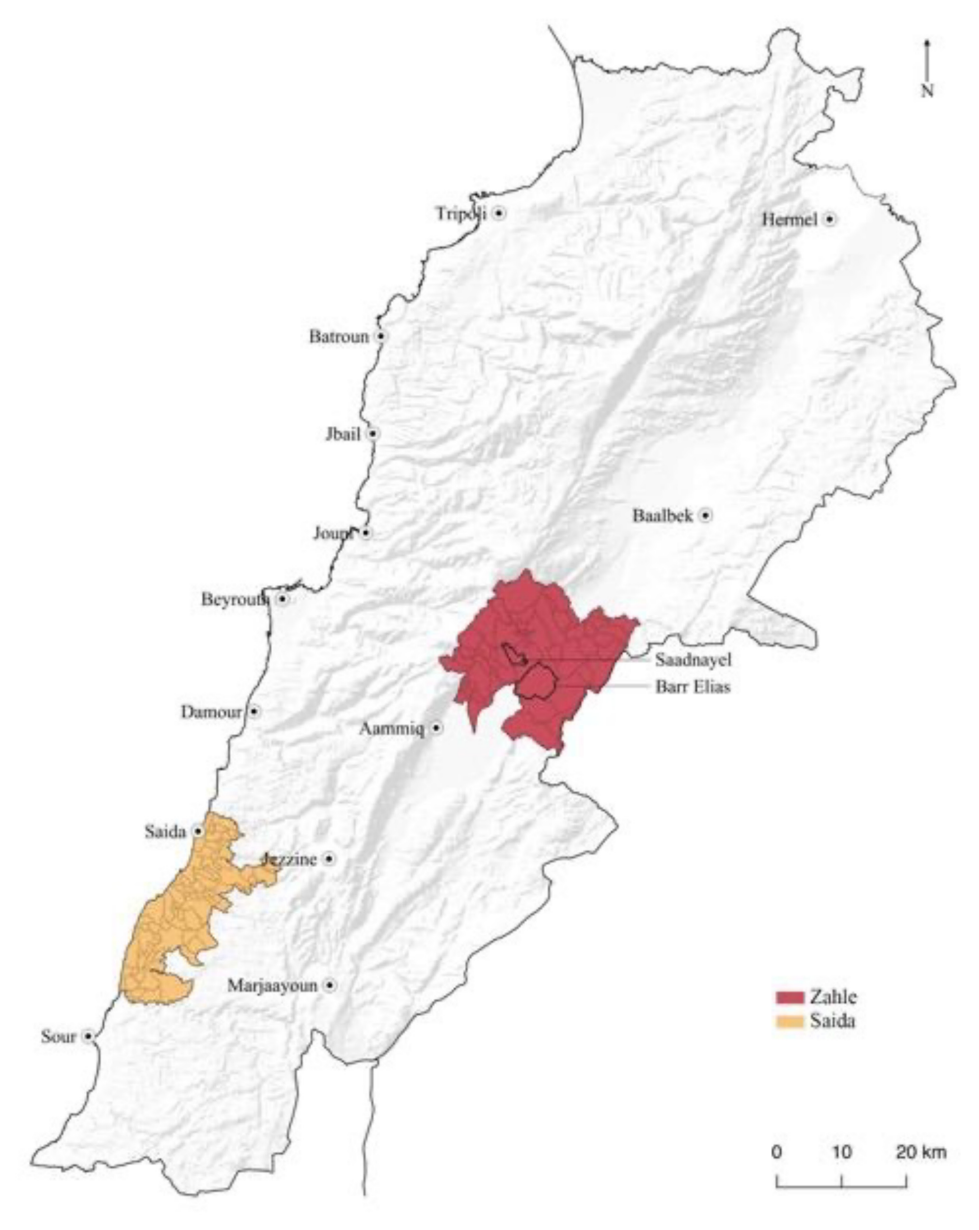
Location of study sites, Saida and Zahle.

A case study approach was employed to study a phenomenon that is difficult to separate from its environment. To grasp the dynamics of the Syrian settings in the country ( Stake, 2005), we explored the displaced everyday lives and subjective experiences of the space in the disparate typologies of informal scape and settlement in Lebanon. The study areas were chosen to represent the three main types of accommodation in Lebanon: informal tented settlement in Bar Elias and Saadnayel in the Bekaa Valley, inhabitation of a non-residential, unfinished building 'The Ouzai Complex' on the outskirts of Saida ( Figure 1), and residential shelter throughout Saida city. These sites were selected based on the extensive research and prior fieldwork undertaken by the project's primary investigator in collaboration with the Civic Centre at the American University of Beirut. The case study approach was based on cartographic interpretation and a series of mapping exercises. It was corroborated by the fieldwork and photographic campaign that the researcher conducted in these locations from 2015 to 2019. This fieldwork aimed at assessing the character and quality of the space in the informal settlings through the recognition of the several forms of appropriation, adaptation, and construction of lived places. Spatial components and uses of the open spaces were chartered to determine relations between inside/outside, public/private, and to understand the everyday rhythms, the activities taking place, the social constraints, the desires of the people, their memories and way of living ( Trovato, 2021).

The desktop analysis and mapping used data retrieved from the UNHCR website. These data were matched with materials from other international and national NGO reports and studies. 

The data collection process in Saida focused on the distinctions between living in a multi-family community-type shelter in the Ouzai complex's unfinished building and living in a single-family unit in a leased residential-type shelter across Saida. Nine out of 10 interviewees had previously lived at the Ouzai Complex but had been evicted by October 2020 and were staying in both residential and non-residential shelters in Saida. In Bar Elias and Saadnayel, eight out of 10 respondents lived in informal tented settlements, while two out of 10 lived in apartment structures.

### Description of the locations

***Bekaa Valley: Bar Elias and Saadnayel.*** Lebanon's Bekaa Governorate is bordered east and west by the Lebanon and Anti-Lebanon mountain ranges. Rashaya, West Bekaa, and Zahle are its three districts. According to Lebanon's Crisis Response Plan demographic package figures for 2020, Zahle has more displaced Syrians than Lebanese, with 243,000 Syrian refugees and 179,000 Lebanese. Most of the non-permanent, tented settlements are situated amid cultivated areas creating discontinuous fringes of sprawled structures in an agricultural context. In the past 12 years, several typologies of tent clusters were erected based on the dimension of the cropped parcels, the presence of natural and built infrastructures, and distance from and relation to the rural villages and cities. Linear informal settlements have been organised along main roads, agricultural channels, and between cultivated fields. Compacted ITSs were located in bigger agricultural plots or on the outskirt of urban settling. Small clusters of Informal tented settlements have developed between greenhouses, scattered and hidden among farming, and adjacent to transit nodes and infrastructures, with walkable distance to employment and productive sources ( Trovato, 2021).

**Figure 2.**
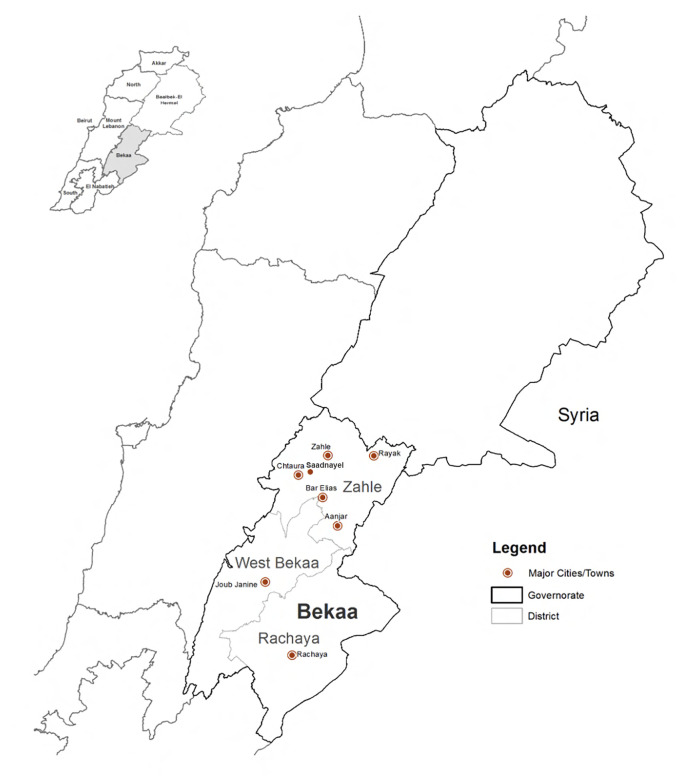
Study area locations in the Bekaa and Saida.

 Zahle's settlements include Bar Elias and Saadnayel ( Figure 2). Bar Elias is 15 kilometres from the Syrian border and has housed displaced Syrians since the start of the Syrian Crisis in 2011. It is home to around 30,000 Syrian refugees registered with the UNHCR. In Bar Elias, there are 219 ITSs, with 52 inactive. The number of tents within the ITSs ranges from one to 203, with a population stretching from 5 to 958 inhabitants. Saadnayel, strategically positioned at the confluence of the Beirut-Damascus highway and the main route connecting the Northern and Southern Bekaa, is home to 17,000 displaced Syrians. Because of its pivotal location, it acts as a transportation and commercial centre, notably for stores and vendors on the international roadway. Saadnayel hosts 90 ITSs, with 20 of them no more active. The number of tents per Informal settlement varies from one to 70, with a population spanning 2 to 425 inhabitants. The informal settlements are mostly concentrated in the suburbs along the national road that leads to the Syrian border. Some of them are hidden between the scattered houses in agricultural fields, but many are visible from the main roads. The organisation of the tents inside the settlements varies from a more uniform rows composition to more organic fabric structures in which adding new space or amalgamation of two or more shelters generates a fluid and informal layout ( Trovato, 2018). The form of the ITSs depends on the dimensions of the agricultural parcels, and site composition could include multiple rows of tents, a square organisation, or a more informal and chaotic dispersal of shelters ( Trovato, 2022a). The ITSs have a relatively consistent typology of room-by-room increments based on space limitations, access to long-span materials ( Dovey, 2013), and restrictions imposed by the owner of the land and by the Lebanese government. Forms of adaptation over time modify the informal settlings' space through generative processes of change. Tents adapt to seasonality, family growth, and changing needs. These adaptive systems are dynamic and based on space and building materials accretions. The typical tents are temporary timber frames covered with plastic, cardboard, and old rugs ( Trovato, 2021).

**Figure 3.**
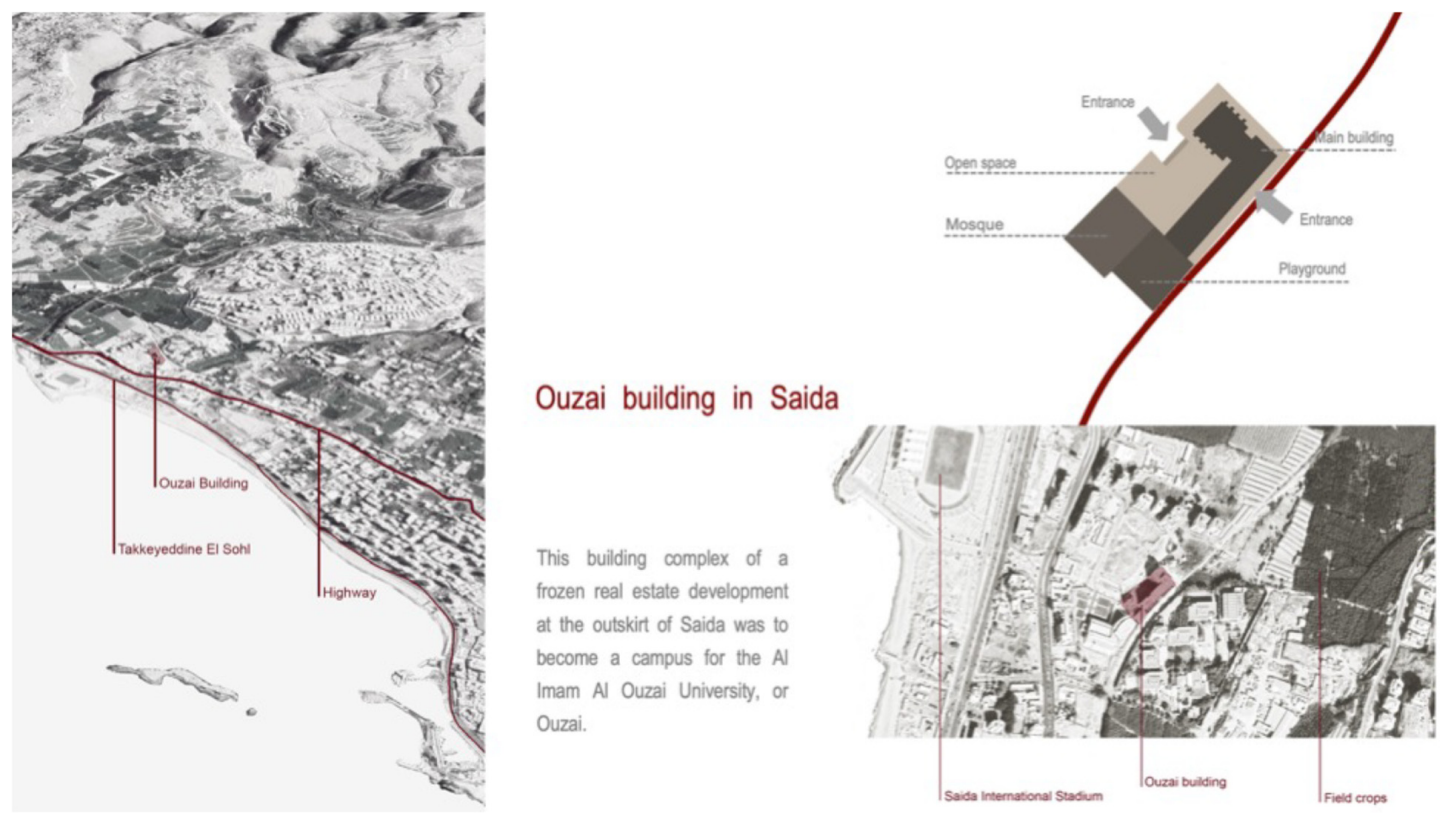
The Ouzai shelter in Saida.

**Figure 4.**
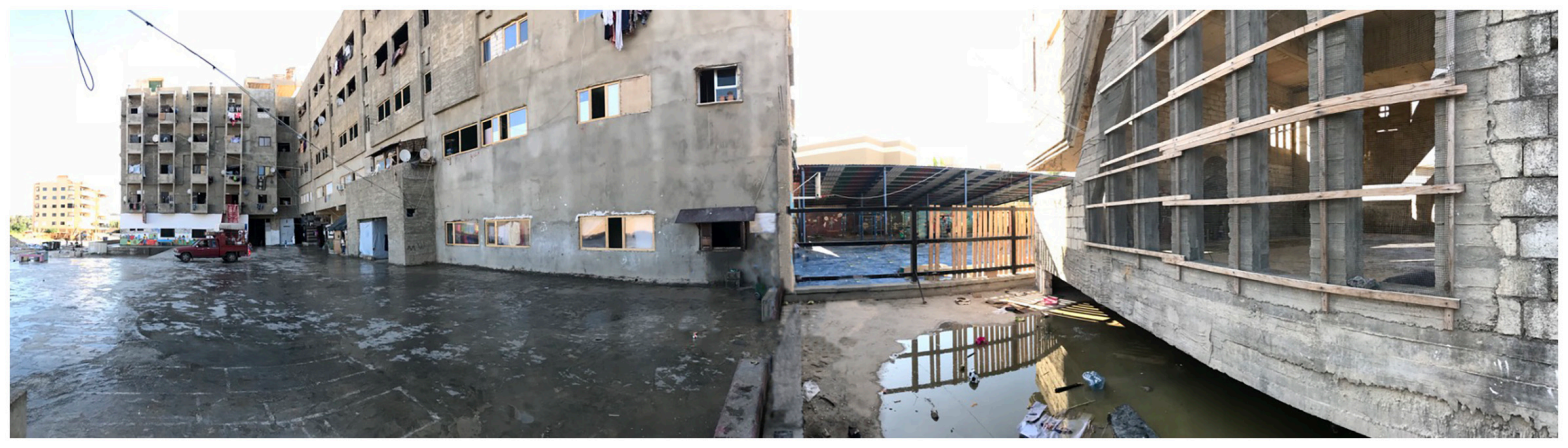
Building façade of the Ouzai shelter in Saida.

***Saida: A non-residential, unfinished building, ‘The Ouzai Complex’.*** People forced to leave their homes and country often prefer to settle in urban areas that offer anonymity, employment opportunities, freedom of movement, access to better services and potential powerbrokers, safety and reassurance in numbers, and strong solidarity between groups of refugees ( Haysom, 2013). Hidden between the urban fabric in the centre or on the periphery of the Lebanese cities, the structures appropriated by displaced Syrians often appeared abandoned and were camouflaged by the generally poor conditions of the surrounding neighbourhood ( Trovato, 2022a). Saida, Lebanon's third-largest city, has more than 1.25 million population. In 2012, the Ouzai complex on the outskirts of Saida ( Figure 3) became home to 180–200 Syrian families fleeing their homes, mainly from Hama, Idlib, and Deir el Zor governorates ( Zaatari, 2015). The Imam Ouzai University for Islamic Studies housed Syrian refugees in an unfinished L-shaped construction with a courtyard and a mosque ( Figure 4). The Saida building existed as a sort of vertical city, so its borders with the urban context have been defined by the marginality in which the structure is located. The informality that characterised this unfinished structure's physical appearance thus expressed the community’s capacity to react to an extreme emergency situation and its constant desire to improve living conditions ( Trovato, 2022a).

**Figure 5.**
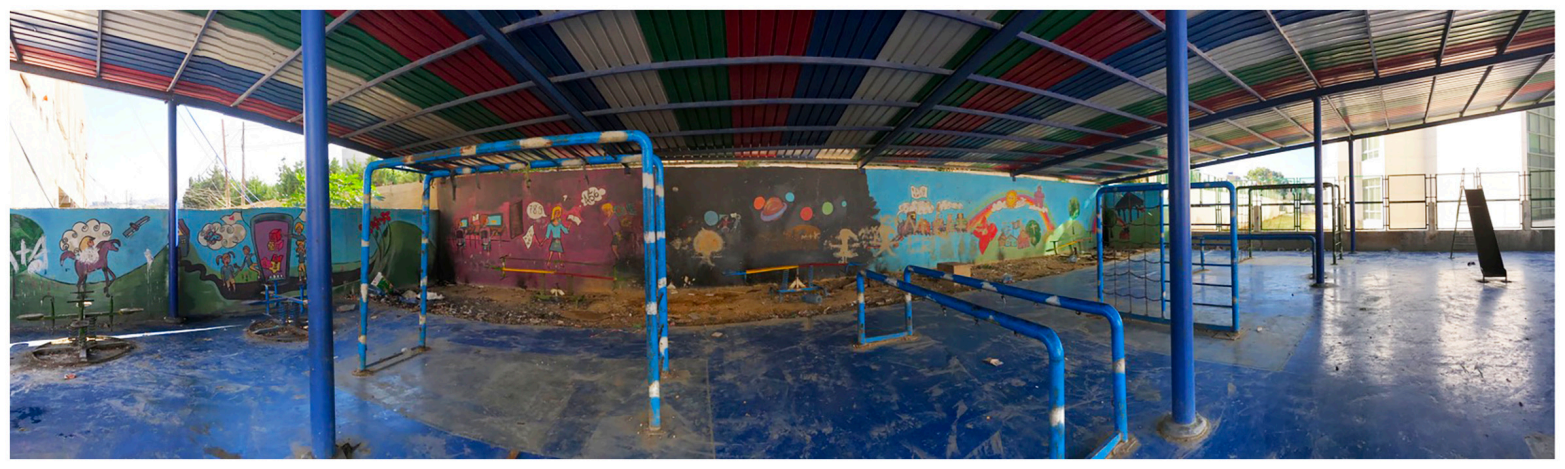
Playground in the Ouzai shelter in Saida.

Syrian refugees collaborated with local and international organisations to make the building habitable by sealing windows with temporary materials, connecting electricity to the complex, and partitioning rooms. Each family would dwell in one room, but the congestion increased with time. Each floor of the building had its own shared restroom. In October 2020, residents of the Ouzai complex were evicted from the building and are now spread around the city, mostly in residential unit shelters. The Ouzai shelter caught several stakeholders' attention since it housed many displaced Syrians in a single compound. Furthermore, the L-shaped structure surrounding the courtyard provided a secure environment for children to play ( Figure 5) while their parents could observe and ensure their safety. Following eviction, most Syrians residing in flats experienced loneliness or a loss of ‘neighbourliness’.

## Results

During the course of the study, we interviewed 209 Syrian refugees in the Ouzai complex in Saida and two informal tented settlements in the governorate of Zahle in the Bekaa region. They used to make their living farming and fishing in rural Syria. There were hardly any regular workers or students among them. The majority took their families with them when they fled Syria, with the goal of eventually settling somewhere in the Middle East. Most of the respondents drove themselves, while the remaining handful walked. The vast majority of the 239 interviewees were either the head of the household or their spouse.

This section of the study summarises the case study analysis and the qualitative and quantitative survey results. It is organised into subsections that comprise our questionnaires' research topics. After providing a general overview of the situation and condition of the displaced in the locations surveyed, the findings focus on the legal status and related issues of protection, aspirations, and priorities of respondents with a gender perspective included, including the types and conditions of shelters depending on the governorate where Syrians are residing, and type of assistance and effects of international development interventions on the displaced communities.

### Respondents’ overall conditions

**Figure 6.**
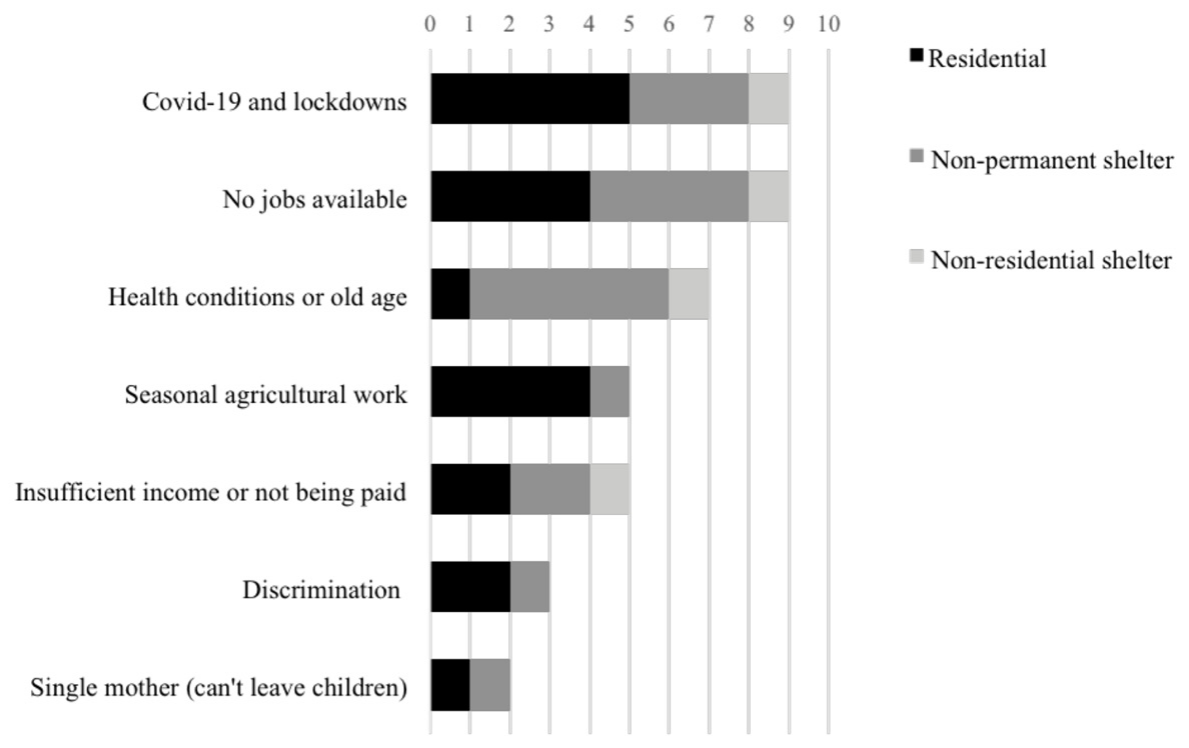
Obstacles finding or maintain employment and the type of shelter displaced Syrians reside in.

Since 2015, Lebanon has received over US$9.3 billion in support for displaced Syrians, vulnerable Lebanese, Palestinian refugees and public institutions under the Lebanon Crisis Response Plan ( LCRP, 2023). However, all surveyed respondents expressed concerns about their living circumstances, ranging from a shortage of daily basics such as food supplies to monthly rent costs and electricity and water bills. Many reported that their situation had deteriorated dramatically after arriving in Lebanon, pointing at the difficulty in the job market as the significant and compelling trigger of their unliveable conditions. Since the surveys were conducted in 2021, COVID-19 was pointed out as the primary reason many individuals were unable to find work and support their families, together with the hyperinflation of the Lebanese Lira that lowered its purchasing power and left many unable to meet their basic requirements. Inability to work was also related to health concerns and elderly age ( Figure 6). “My living conditions are pretty bad now; it's bad for Lebanese people; what would Syrians say?” commented Participant AG.

### Respondents’ legal status and protection

As well explained before, the Lebanese Syrian legal status evolved from the beginning of the war till today. Syrians escaping the conflict are not recognised as refugees but are labelled as displaced. In our study, we found out that except for one respondent in Bekaa, who didn't have legal documents and depended on his wife and children's registration for help, all respondents in Saida and Bekaa were registered as refugees with UNHCR. At the time of the survey, the majority of registered respondents were under international aid support and reported that it was "not enough" to satisfy daily requirements since "everything is pricey presently."

**Figure 7.**
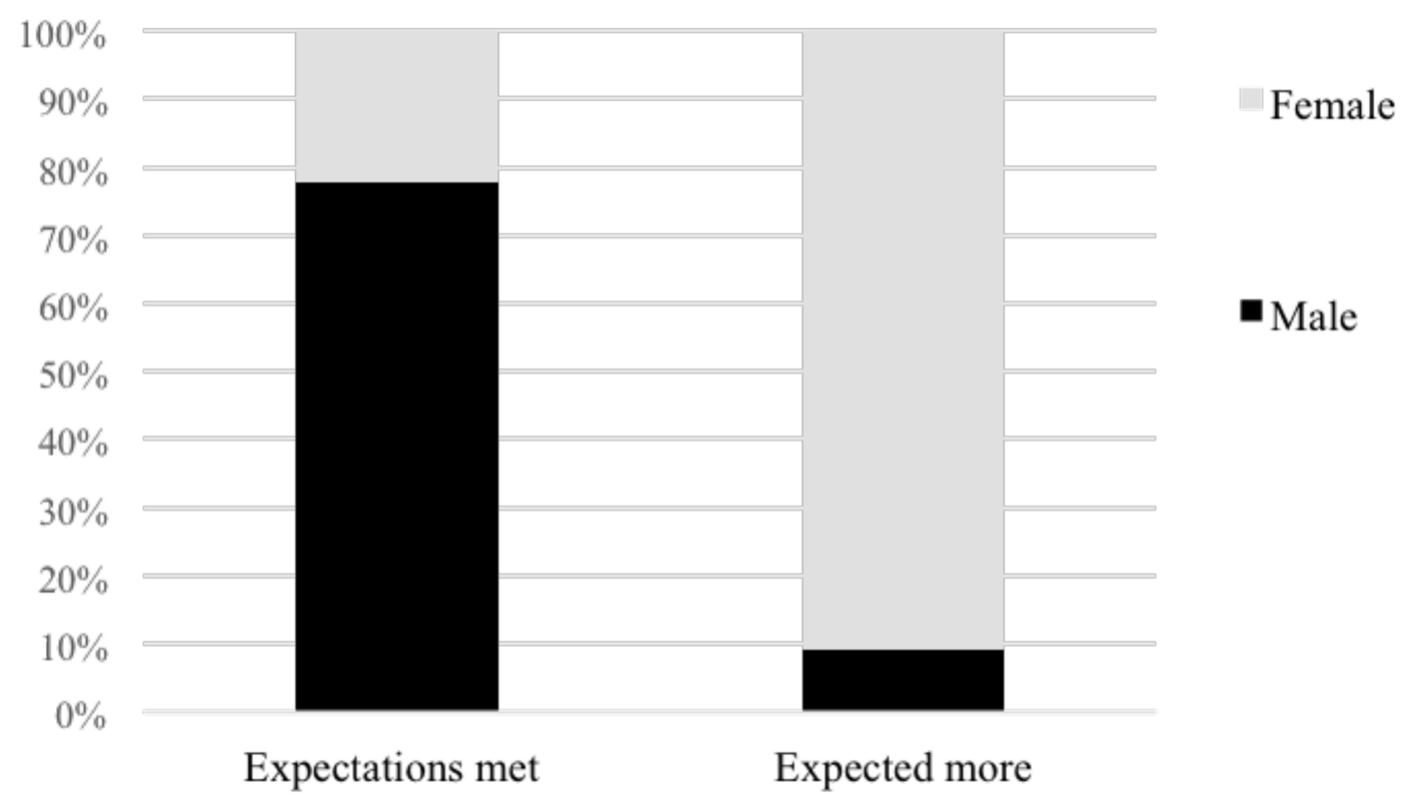
A gender analysis of whether expectations were met registering as a UN asylum seeker.

A gender study of whether respondents' expectations were fulfilled by their refugee registration revealed that female participants were usually more unsatisfied with the results, with most of their expectations not being realised ( Figure 7).

As refugees registered with UNHCR, the majority of respondents did not perceive a change in their identification. A minority, though, felt sceptical about their new status. Respondents saw themselves as refugees, uprooted, insecure, and exposed to the whims of authorities.

“I feel like a refugee, not in my own country. I have not been settled, and I have not received my rights.” Participant 19
“My situation has implications. My wife and I are ill and require medical attention. I might have gotten that medical help if I hadn't been a refugee.” Participant Z

On the other hand, the impressions and repercussions of possessing a residence permit received mixed evaluations. Some thought it gave them a feeling of safety, security, and protection from Lebanese authorities' inquiries, while others' expectations were not satisfied. Respondents without a residency permit/legal documents, on the other hand, often avoided travelling to isolated locations or leaving their place of residence entirely, fearing imprisonment by authorities.

“Now I'm at ease. It is safe to have a residence permit. Now I feel like someone oversees me.” Participant 16
“I assumed that after we got our residence status, our quality of life would improve, and we'd get more contributions. But it did not occur. In truth, our situation is worse with time. “ Participant 244
“The Lebanese General Security Service has custody of my legal papers (ID). So I can't leave the house because I'm frightened of the checkpoints. I might go to prison if they catch me. How will I feed my family and pay my rent if I can't go out?” Participant 51

### Aid and supplies

The prolonged nature of the Syrian crisis prompted a shift from an early emergency reaction to a development-oriented, long-term response, which required coordination among the many humanitarian players on the ground. The Lebanon One Unified Inter-Organisational System for E-card (LOUISE) platform was created in 2016 to manage sectoral and multi-sectoral cash and voucher aid. This enabled the World Food Programme, the UN Refugee Agency (UNHCR), and the United Nations International Children's Emergency Fund (UNICEF) to transfer funds to Syrian refugees using a common e-card. The Multi-purpose Cash Assistance Programme (MCAP) identified and assisted disadvantaged families by using both the UNHCR database and the Vulnerability Assessment of Syrian Refugees (VASyR) ( UNHCR, 2021). A winter aid program, the Protection Cash Assistance Program (PCAP), and Emergency Cash Assistance are also available (ECA). MCAP recipients spend most of their funds on food, housing, cleanliness, and healthcare ( UNHCR, 2021). Nonetheless, nine of 10 Syrian families lived in severe poverty in 2020 and 2021. ( UNHCR *et al.,* 2021).

According to our results, all respondents in the Bekaa were getting financial contributions at the time of the interviews. In fact, most responders relied entirely on these gifts to cover their monthly costs. Furthermore, almost half of those polled had received UNHCR and other non-governmental organisations (NGOs) supply gifts such as construction goods, including nylon coverings, wood, or roofing material, hygiene kits and furnishing items such as beds and pillows. There was a significant discrepancy in the respondents' experiences in Saida. Only five out of 10 respondents were receiving cash donations when they were interviewed, although the majority of those who were not receiving aid were living in very difficult circumstances, were indebted to landlords or local grocery stores, and had repeatedly contacted UNHCR to review their case, hoping to be eligible for cash assistance. Respondents who did not receive financial aid had previously had supply donations, such as food boxes, hygiene kits, and furniture goods while living in the Ouzai complex. This mismatch between residing in community complexes (Ouzai complex) and camps vs residential shelter type may be attributed to the Lebanese Government's no-camp policy. While the international world commended Lebanon's open border policy and non-encampment policy during the first phase of the Syrian crisis, research has shown that camps facilitate the delivery of supplies and the effectiveness of humanitarian services ( Turner, 2015).

In Saida, a single mother explained:

“I haven't gotten any assistance or donations from any organisation since I left the camp (Ouzai complex), and it's been almost five months.” Participant 243

A married man in the Bekaa shared:

“All the help goes to the mokhayam (informal camp) [...] Until today, I have not received any packages since 2013. They provide hygiene equipment, but I am not getting any since I live in a rental property.” Participant B

Several respondents raised concern about MPCA's unreliability, claiming that monetary contributions may cease at any time and that they needed to know when or why their cycle would begin or end. One responder said:

“I used to get aid from the omam (UNHCR), but they stopped assisting me for four years without explaining why, and now they're back. Thank you, God.” Participant 8

### Shelter: situation and structure

From 2016 to 2020, the shelter sector had a funding shortfall and a continuing drop in money compared to the sector's appeal ( UNHCR & GoL, 2020). The shelters are classified into three types based on the UNHCR *et al*. definition (2021): residential, non-residential and non-permanent. Residential shelters include apartment buildings/houses, concierge rooms or hotel rooms, and are meant for people to live in. The non-residential shelters are defined as something not intended for dwellings but have been appropriated as such. Examples are unfinished buildings/active construction sites, factories, workshops/warehouses, farms, or engine/pump rooms. Tent dwellings and prefabricated units are examples of non-permanent housing.

**Figure 8.**
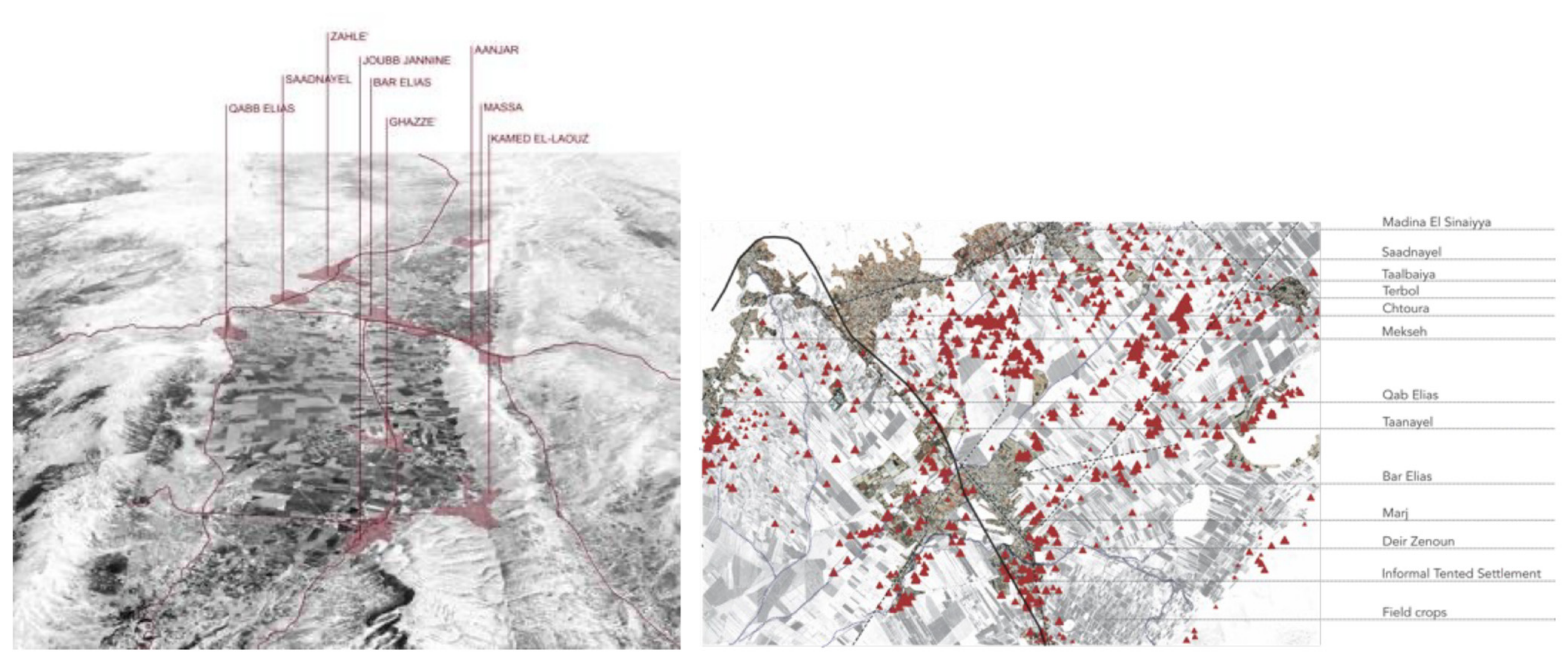
The Bekaa Valley and Informal Tented Settlements.

**Figure 9.**
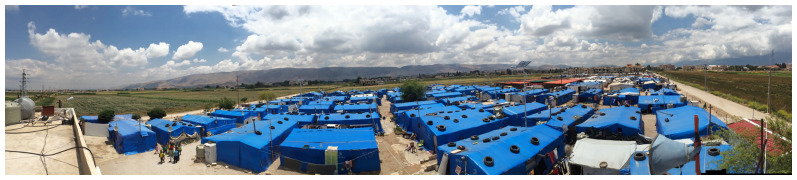
Bird-eye view of an Informal tented settlement in Bar Elias.

***Bekaa Valley: Bar Elias and Saadnayel.*** Most displaced Syrians in Bar Elias and Saadnayel reside in agricultural fields ( Figure 8), near public and private schools, informal vocational facilities, municipal offices, and the primary healthcare facility ( DeJong *et al.,* 2017). Landlords in Bar Elias are more inclined to convert their agricultural properties into informal tented settlements ( Figure 9) to be rented by displaced Syrians since this is considered more profitable than farming ( Al Ayoubi, 2018).

**Figure 10.**
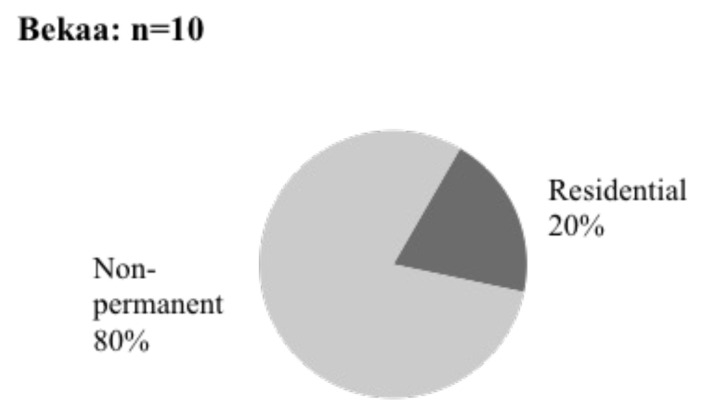
Shelter types in the Bekaa Valley.

As a result of our qualitative study, eight respondents out of 10 lived in informal tented settlements in the Bekaa Valley ( Figure 10). The remaining two responders resided in Saadnayel and Bar Elias residential buildings.

**Figure 11.**
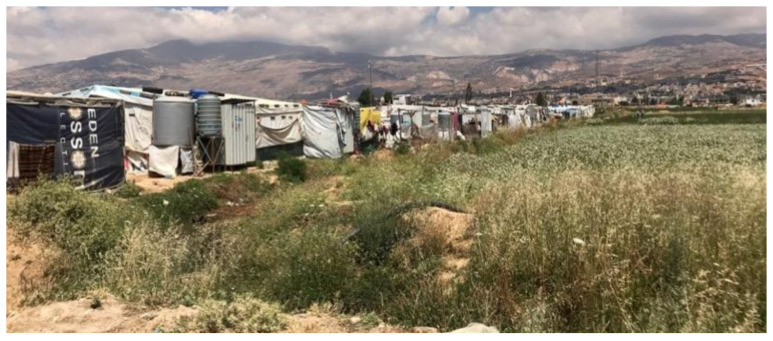
Informal Tented Settlement in Saadnayel.

**Figure 12.**
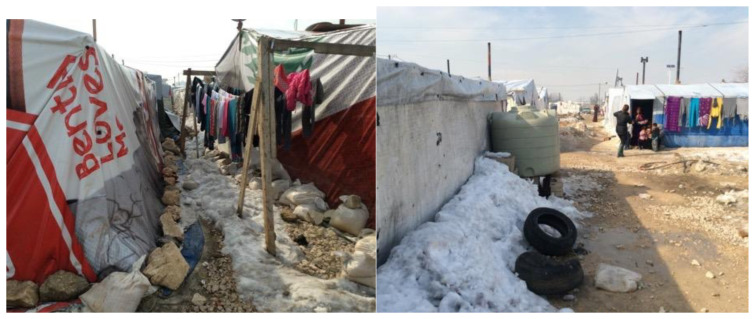
The Informal tented settlements' conditions in winter.

Informal tented settlements have encountered issues such as garbage heaps due to an inadequacy of a solid waste management system, a lack of drainage and sewage systems, and poverty in housing construction materials to resist the Bekaa Valley's harsh winters ( Al Ayoubi, 2018). Most of our respondents expressed concerns about the tented shelter's lack of structural integrity and drainage and sewage facilities. Tented shelters are made of wood and nylon sheets, and tires are often used to hold the roofing in place. Generally, this cannot withstand storms, and roof leaks were a common complaint. To guarantee the health and well-being of the displaced people, the Bekaa Informal Tented Settlements often rely heavily on help from NGOs and Water, Sanitation and Hygiene (WASH) programs. However, in Saadnayel, drainage issues have arisen, particularly during the rainy season, when septic tanks overflow and flood the tents. Overflowing toilets and lacking sewage infrastructure mostly resulted in poor sanitation and hygiene ( Figure 11). Due to a lack of drainage, tents flood, causing disease among residents, damaging furniture, and forcing families to live together while tents are emptied of water, resulting in congestion and tension. More than half of those polled said the winters are too cold, and they didn't have enough money to heat their houses or keep their children warm ( Figure 12). In the Bekaa, each family unit had more children than in Saida, with an average of six kids in the Bekaa vs four in Saida. This frequently made it more difficult for parents to care for their children and have enough goods to keep them warm throughout the winter.

“Now that it's winter, the tent floods when it rains, and I don't want to go to my brother-in-law's home because my children's sounds would upset him.” Participant AG
“I didn't get the UN's fuel supply this year, so I wrap my kids in blankets when they're cold since I can't afford to purchase diesel.” Participant AG

Poor living circumstances have led many owners to quit their tents in Saadnayel and Bar Elias, and displaced people are looking for alternatives that might suit their requirements ( Najjar, 2019).

**Figure 13.**
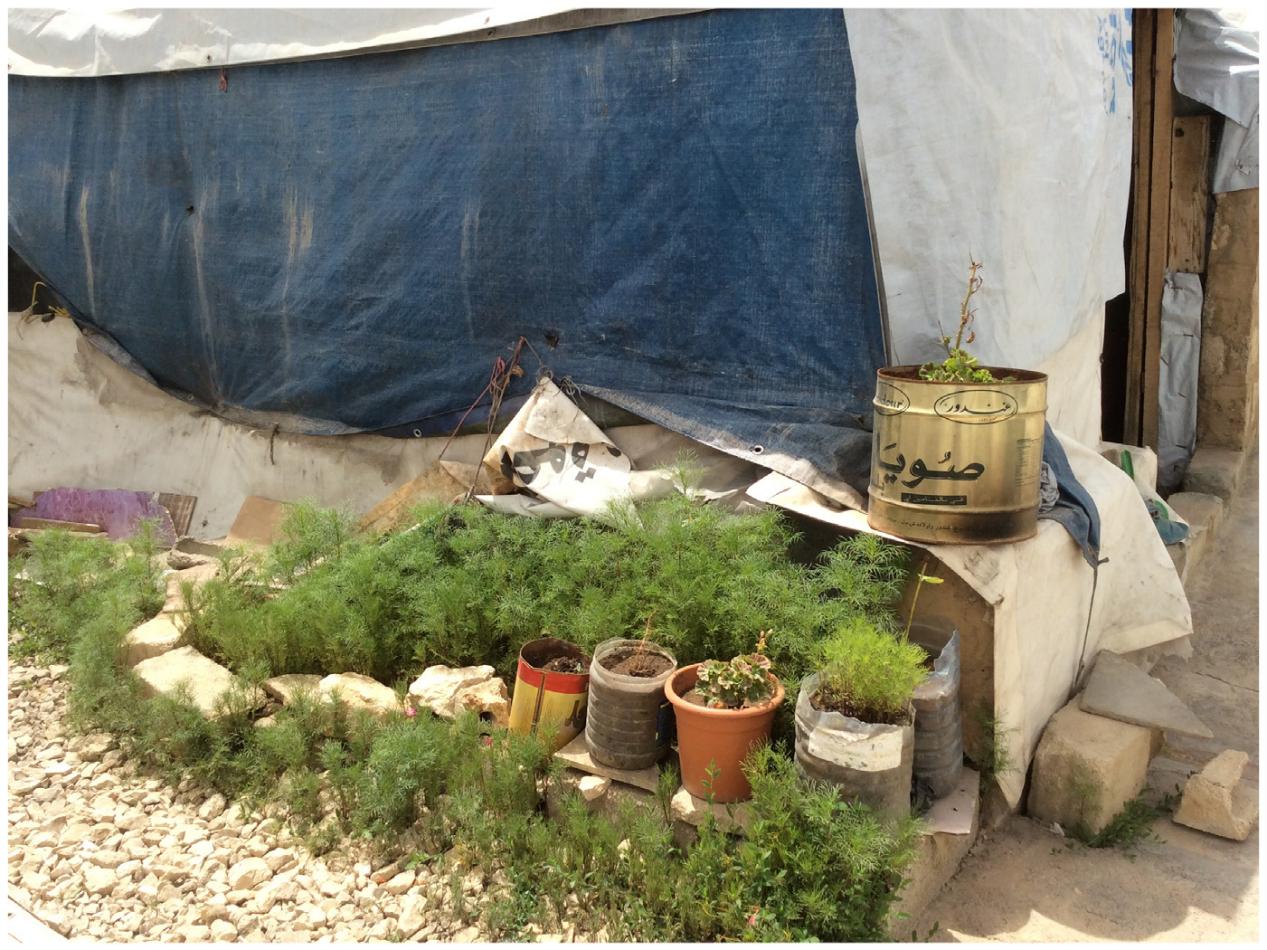
Community spaces in the Informal tented settlements.

Inside the informal tented settlements, community spaces are informal and are often handled by individuals. They may incorporate mobile chairs, herbs for food gathering, or shade plants to shield from hot weather, much like pocket gardens ( Figure 13). These areas are often fleeting and deteriorate due to seasonal changes or neglect. Almost all respondents were aware of their ITS neighbours and their numerous familial-like relationships. Only three respondents stated they do not mingle with them regularly and saw them as acquaintances; one said they had no contact with them. Most Syrians living in the Bekaa’s ITSs, visit, interact, and share meals with their neighbours regularly. With a fragmented governance structure of displaced Syrians in Lebanon, state actors such as municipalities have had the authority to impose haphazard curfews that limit displaced Syrians' movement and mobility. Additionally, non-state actors such as landlords and security guards have used their power to regulate gathering spaces and access to services and carry out raids or evictions ( Fakhoury, 2020). Bar Elias has received evicted displaced individuals from neighbouring localities like Zahle ( Al Ayoubi, 2018). Since the outbreak of COVID-19, situations for displaced people in ITS have become difficult. Due to the lockdown, travel limitations, and social distance measures, displaced people, particularly vulnerable groups with health issues and needing frequent health supplements, have not received critical help from NGOs. In addition to the lockdown and curfew, the municipality of Bar Elias prevented displaced people's mobility by requiring displaced individuals to choose a representative of the community to ensure the ITS’s basic requirements and tell dwellers of its decision ( HRW, 2020b). Respondents expressed difficulties finding work as a result of both COVID-19 and the economic condition, making it difficult to make ends meet.

**Figure 14.**
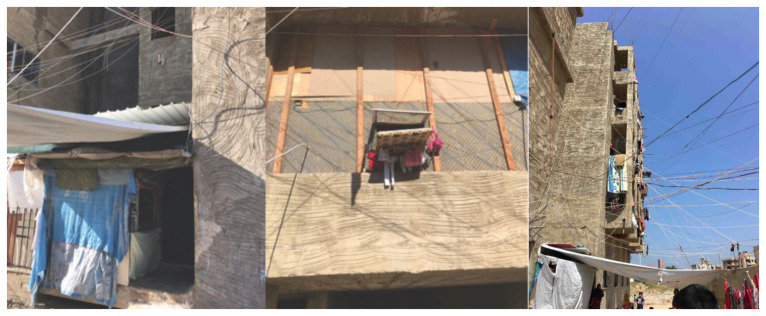
Temporary closures and windows in the Ouzai complex in Saida.

**Figure 15.**
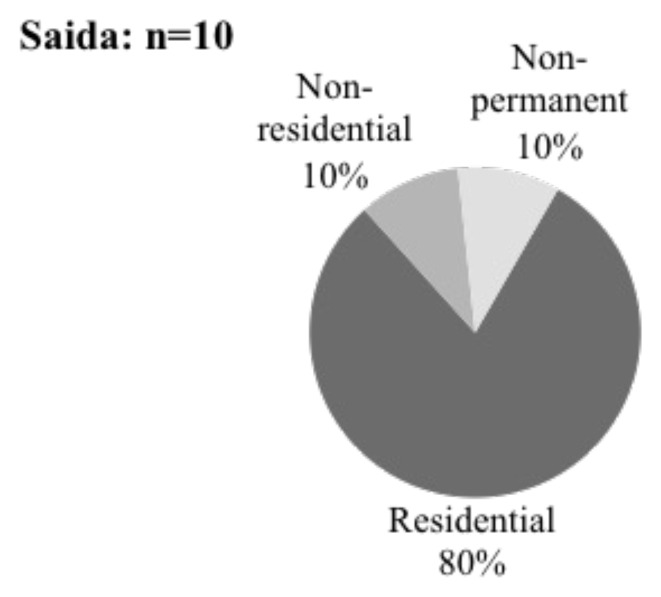
Shelter types in Saida.

***Saida: A non-residential, unfinished building, ‘The Ouzai Complex’.*** Before the eviction, the complex was unique since it housed displaced people with family and local links, resulting in a communal and safe environment ( Figure 14). During that period, there were few problems with the Saida population, and several Syrian-owned businesses were set up on the bottom floor of the Ouzai refuge ( Zaatari, 2015). In 2015, displaced Syrians risked eviction because the university's owners sought to regain their property and complete the construction (ibid.). However, evictions were delayed when the Ministry of Social Affairs intervened and reached an agreement with the owner, allowing displaced people to stay. After being evacuated in October 2020, displaced Syrians struggled to secure new housing. Many live in flats surrounding Saida, straining to make ends meet with rent and utility bills. They did not previously pay significant rental rates but now face monthly rent payments ranging from 250,000 LBP to 600,000 LBP. Some respondents covered their rent requirements by paying out of pocket, while others utilised UN cash assistance. Over half of those evicted from Ouzai stated they looked for and found their present housing independently, with some using previous neighbours' recommendations on the lowest rent. As a result, 8 out of 10 respondents lived in residential buildings, one was residing in a non-residential structure, and one was in a non-permanent shelter in Saida Informal settlement ( Figure 15). Since leaving the Ouzai refuge, Syrians residing in flats in Saida have faced additional obstacles, including exorbitant expenditures.

Since their evictions, respondents agreed that, although the move was expensive, it provided more opportunities for social distance than their former way of life in the compound. COVID-19, on the other hand, as well as the economic environment, have reduced work options. Although paying rent and meeting power and water expenses constituted an extra cost for many respondents, the majority expressed satisfaction with their increased privacy, structural safety, and better living conditions in the apartment. Unlike at the complex, where children often spent their days in the courtyard with many other children, respondents were now pleased to be always close to their children, ensuring their safety. A single mother who currently lives in an apartment has the following:

“We didn't have to pay rent before, but what about the conditions? The rental is preferable since it is sheltered; formerly, we [family in the Ouzai complex] lived in one setting. My children are always protected here.” Participant 8

A male apartment dweller said,

“I like this house because it has privacy, closed windows, more than one room, and is simpler to winter. All that counts in this home is paying the rent and the power bill.” Participant 1

While some respondents were relieved to have more than one room now that they were living in flats, one was dissatisfied since she didn't have a kitchen and had to repurpose her balcony as a kitchen. Indeed, landlords sometimes partition properties and rent them to Syrians, resulting in a lack of services and infrastructure in each residential unit. One female respondent live now in a non-residential, unfinished, and abandoned structure in Saida. Those structures, like garages, factories, workshops etc., are unsuitable for living and critically substandard, lacking infrastructures and services. Our respondent reported that she used to take her children down to the basement of the building when it was rainy or chilly. She expresses herself as follows:

“My challenges remain in my home since it is not a true house. You do not feel secure. The sun does not come in. It's always dark here. I feel sad for my children since they must live in such conditions.” Participant 19

### Community, hospitality, and pride

The majority of respondents in the Bekaa and Saida felt safe in their neighbourhood, two disliked going out at night, and just one (a single mother) did not feel safe in her home or neighbourhood. The majority of responders in the Bekaa get assistance from their community, including neighbours, family, friends, or the shawish (camp supervisor). Only one responder said there was poor treatment in the ITS and that she did not get 'complete packages' of contribution boxes since residents often removed them from neighbouring or inhabitants' informal settlements. While communal life worked best in the ITS, some parents found it difficult to accept their children being mixed in with others. Squabbles between children often escalated and reached the parents, breaking relationships. 'I don't want to live in a camp where my kids would interact with other kids and the surroundings in the camp,' says a resident of an apartment complex in the Bekaa.

In Saida, there were mixed feelings on community assistance: four out of ten respondents thought the community was beneficial, two thought it was neutral, and four thought the community was not helpful or had no relation. For Syrians living in flats, commonplace hospitality customs are becoming more difficult to sustain. Relationship development among Syrians is sometimes hampered by a lack of resources to assist or host individuals. A single mother explained:

“I can't afford to invite anyone to my place right now, and I can't even afford to give them coffee. That's why I don't form ties with anybody, so no one comes to see me.” Participant 243

Another single mother expressed her thoughts about settling in Lebanon:

“The environment here is different from that of Syria. In Syria, we have rituals that help new neighbours settle in, cook for them, and make them feel welcome. These customs do not exist in this country. Nobody has ever done it to me.” Participant 16

However, a male apartment dweller has favourable feelings about his neighbourhood, including Lebanese and Palestinian neighbours:

“All my neighbours are Palestinians and Lebanese; we have extremely amicable ties. As Syrians, we are a hospitable and open community, tightly knit; we live with our family.” Participant 1

### Aspirations and priorities of respondents

**Figure 16.**
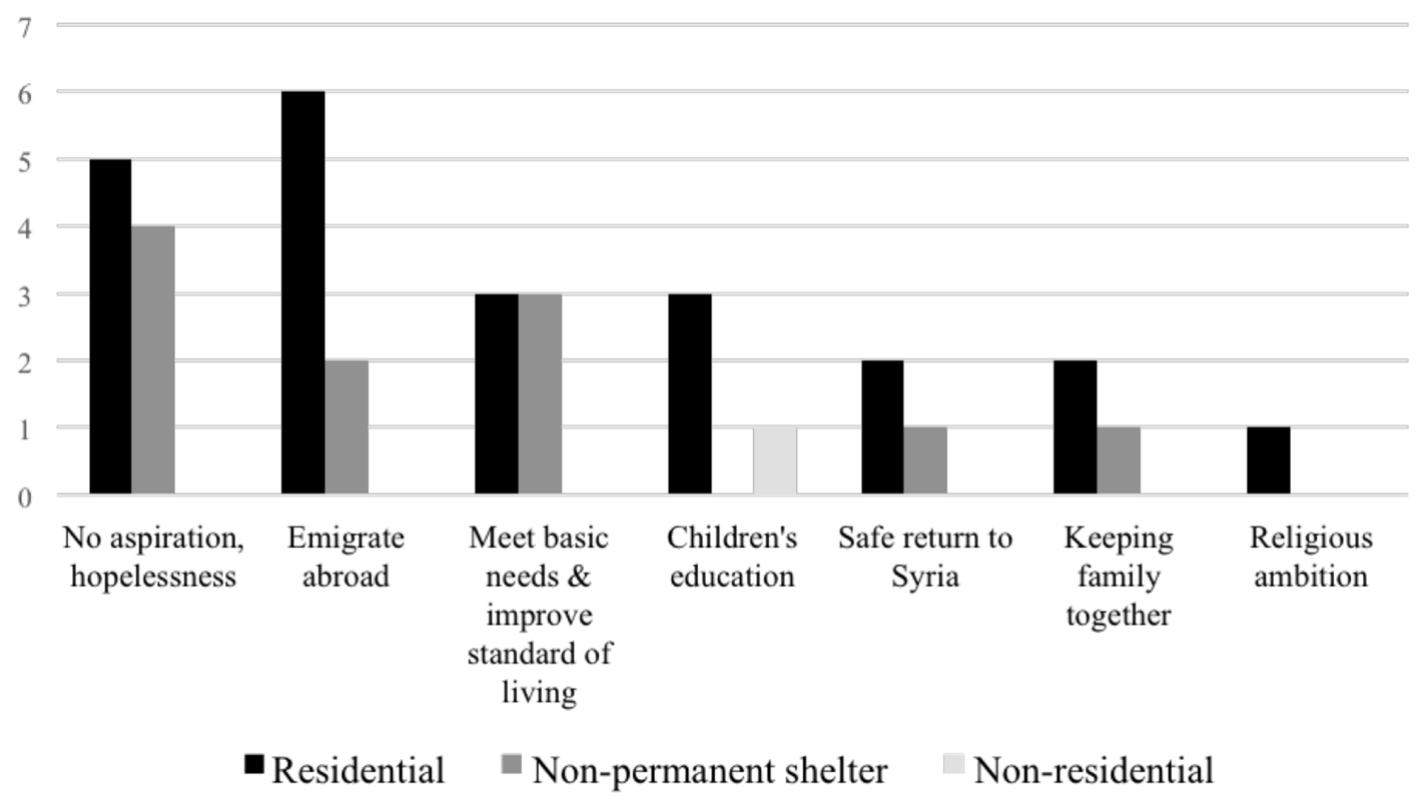
Syrians displaced dreams or aspirations since settling in Lebanon between ITSs, apartment buildings, or informal buildings.

Respondents at residential shelters reported the strongest desire to immigrate to another country ( Figure 16). In tented non-permanent shelters, interviewees shared this desire; however, the majority were gloomy about the future and just wanted to meet their current needs and improve their quality of life. A prominent concern of Saida parents, which Bekaa respondents did not share, was that their children were not obtaining an education, which was their ‘right’. Despite the free education, respondents in Saida reported they couldn't send their children to school due to hefty transportation costs. A single mother living in an apartment in Saida discussed her concerns about dropping her daughters off at school in the afternoon:

‘Right now, all I want is for my children, like other children throughout the globe, to obtain an excellent education. I can't afford to pay for the school bus, and my kids go to school in the afternoon, so I don't feel safe letting my daughters remain out late at night.’ Participant 16

### Female respondents: hardship of single motherhood

The majority of females interviewed in Saida and Bekaa were single moms. Their spouses had either abandoned them and remarried, or they had gone missing after being detained at the border or in Syria. Being the only provider for the home as well as the sole caregiver is extremely tough for single mothers. Several moms voiced their dissatisfaction at being unable to leave their children alone while going to work. Single moms in Saida claimed they were not getting UNHCR monetary or supply assistance even though they did not have a partner to support them. Some respondents said they were just ‘weary’ and couldn't deal with the issues in their life. With limited choices, a single mother in Saida intended to supplement her income by having her 12-year-old kid sell gum on the streets. Another single mother expressed her despair and desires to build relationships:

“I want someone to knock on my door and ask us how we’re doing, I don’t want money as much as I want someone to empathise with us.” Participant 36

Respondents in the qualitative interviews agreed that no development initiatives impacted their migration ambitions. On the contrary, the absence of such measures compelled individuals to consider leaving Lebanon or returning to Syria.

“No, there haven't been any interventions to persuade me otherwise." The living conditions are reason enough for me to go. It is a long-term choice.’ Participant 51

Except for one who managed to contact an embassy for the visa, none of the responders planned for their future. A lack of preparedness resulted in a lack of knowledge, access to information, and people to contact. All participants said that they needed the UN's help in making decisions. Furthermore, no one attended any training sessions for this purpose.

“No, since I'm at a loss for what to do. I want the UN to help me with my migration.” Participant B1

Furthermore, although a small percentage (7%) had job business ambitions such as farming, the majority (93%) had no choice. Furthermore, just two respondents desired a family reunion.

“I'm hoping to be able to work. I don't have a preference. I can work everywhere as long as I can provide food for my children.” Participant 29
“I wish that I would have the chance to travel outside of Lebanon, because in here, I’m not receiving my rights.” Participant 244

Finally, when asked to propose ways to enhance their quality of life, several suggested lowering the USD: LBP currency conversion rate, expanding access to essential goods, and increasing work prospects. Furthermore, some advocated for additional organisations to assist refugees, while the majority saw little hope for a brighter future for Lebanon.

“I don't want to enter politics, but the dollar's value should fall so people can purchase food and survive.” Participant 5

The results of our data analysis show that the development interventions and aid assistance, even if necessary for survival, didn’t meet the expectations and didn’t enhance the quality of life of displaced Syrians in Lebanon.

## Discussions and Conclusion

Our analysis of Syrian migration in Lebanon found a gridlock in the displaced community's condition. In the past twelve years, the GoL didn’t succeed in assuring the respect of basic human rights and a sufficient quality of asylum. Legal protection for displaced Syrians lacks a formal domestic legal framework or laws that could guide authorities and humanitarian actors alike. Undoubtedly, the emergence of the COVID-19 pandemic and the 2020 Beirut Port explosion have exacerbated the already deteriorating country's economic situation, causing waves of extreme political unrest and government resignation. However, the lack of a formal and legal asylum framework jeopardised the protection of displaced Syrians from access to basic needs, legal status, healthcare access, and proper accommodation. Having unresolved residency status contributed to increased vulnerability to exploitation, high poverty levels, and child labour. Displaced Syrians' uncertain legal status also fostered feelings of uneasiness, notably when authorities conduct random searches at checkpoints, and they lack legal documentation, namely the Lebanese residence permit. Although this research did not concentrate on gender, it is essential noting the disparity in legal standing between Syrian men and women. While in 2020, the number of women without legal residence remained unchanged from 2019, the lack of legal residency inhibits women from reporting incidences of harassment or assault. Syrian women are, therefore, more vulnerable and prone to assault and prejudice ( VaSyr, 2020). More than half of the female participants were single parents, either widowed or separated, or the whereabouts of their spouses were unknown. Being alone amplified their anxiety while striving to satisfy basic requirements and balance jobs with duties for their children at home alone. It has also been upsetting for Syrian women not to know where their husbands are or how they are doing.

Having an asylum seeker status impacted the perceptions of a little less than half of our respondents, who reported feeling like a 'refugee', 'uprooted', 'vulnerable', and 'without a home'. Displaced Syrians with a residency permit reported feeling 'safe and secure' and protected from authorities' arbitrary search and investigations. However, attaining a residency permit proved difficult for the majority of the respondents, where changes in Lebanese laws and costly fees were communicated as the main obstacles to acquiring it, confirming VASyR findings. Illegality may result in high penalties, feelings of insecurity, and threats of evictions or labour crackdowns without notice. Illegality also limits people's freedom of movement and independence, restricting access to healthcare and opportunities to change legal status and increasing their reliance and dependency on community, government, or informal assistance networks and aid mechanisms. Most of our interviewees received development interventions, including formal and informal support. Basic assistance (cash, vouchers, e-cards), food security, and health and nutrition were assured to our participants in the qualitative interviews, but they didn’t meet Syrians' expectations or improve their quality of life. The main criticisms encompassed inadequate financial assistance (considering the devaluation of the Lebanese Lira) to meet basic daily needs, lack of aid in education and health services, and lack of aid in migration services or migration information. Besides, the disparity in assistance between urban and rural locations appeared evident, with the cities receiving less support due to the challenges humanitarian relief and support providers may have in reaching displaced Syrians in urban regions. Unlike ITS, the majority of displaced Syrians living in apartments are not receiving any assistance from the UN, although they must meet the costs of rent, and many are falling into debt with the owners, friends, and family. Participants reported using other UN assistance (i.e., diesel winter assistance) or remittances abroad to pay for rent. Furthermore, those evicted from the Ouzai shelter didn’t rely heavily on food assistance from the UN. Instead, they could only buy the cheapest necessities out-of-pocket or borrow money from shop owners, family, or friends.

In addition, humanitarian assistance services were often duplicated, resulting in some popular ITS receiving multiple donations at once while others were neglected. Respondents have repeatedly manifested frustration and bafflement about the UN's decisions over aid or assistance distribution. The patchy distribution and the reasons for aid suspension or reinstatement have left many Syrians bewildered. Frustratingly, there was no clear explanation of who qualifies for assistance and who does not. Therefore, it would benefit Syrians to have clear and complete information regarding UN support or service.

The displaced, on the other hand, lean on social and personal networks, including family, friends, and neighbours, to navigate through difficult times. In contrast to Saida, community relations in the Bekaa are noticeably more positive. Most Bekaa residents said they relied on one another and on community members to get through tough times. However, unlike rural settling, most Saida residents felt lonely. Despite family and community ties, most displaced do not financially support one another since they all share similar precarious conditions. Most Syrians' primary source of protection from the difficulties of life in Lebanon is their families. But there were reportedly problems within the family unity. Nearly half of those polled recounted struggling to meet their children's fundamental needs, notably nutrition. In fact, the dire economic situation in Lebanon has made it difficult for Syrian parents to provide basic necessities like diapers and milk for their children.

Furthermore, the country's multiple crises are causing personal and emotional stress and exhaustion. Syrians described feelings of mental fatigue, tiredness, emptiness, pessimism, desperation, and demoralisation. Before the Syrian conflict, many displaced Syrians in Lebanon reported several hopes and life objectives they had (and attained) in Syria. These included (in descending order of importance) ensuring a happy and prosperous future for their family; ensuring their children received a good education and were raised well; purchasing some land and/or building their own house; harvesting their own farmland; and maintaining a decent living with good financial stability. However, many of these aspirations were dashed with the outbreak of the war. When asked what they thought would help them face their issues, Syrians mentioned that consistent, succinct, and transparent information on UN support and/or aid would benefit them.

Unfortunately, COVID-19, the Beirut blast and the economic crisis have made it difficult for many to retain or form new social networks since too many displaced people experience comparable challenges and cannot turn to others for assistance. Consequently, Syrian displaced living condition has deteriorated, with the vast majority still in perilous conditions and below the poverty line, with nine out of ten living in extreme poverty ( UNHCR, 2023b).

Due to the absence of protective measures and development aid, Syrians displaced future in Lebanon is uncertain. In fact, 62% of Syrian refugees have expressed a wish to either settle in a new country or return to Syria, a significant rise compared to 2018. ( WFP, 2020). First and foremost, people chose to relocate or return to have a better quality of life, adequate access to education and healthcare facilities, and to be closer to their extended families already living in another country. People mainly decide to stay because they are either waiting for the situation in Syria to improve, allowing them to return, lack of money or knowledge about migration options, are single mothers, or are old.

When comparing 2021 migration aspirations to projected aspirations, there was an increase in those who "don't know" their migration intentions, and 93% of participants reported having little control over their lives. The qualitative research also found out that many displaced Syrians thought they had no choice about their future plans. They felt trapped because of the country's situation, COVID-19, the ongoing conflict in Syria, and a lack of knowledge regarding migration or recruiting procedures. This validated secondary resources, whereby displaced Syrians thought they were kept in "limbo" ( RPW, 2020). Most respondents said the situation in Lebanon and Syria caused them to rethink their travel plans. Others claimed to base their migration decisions on second-hand information and experiences from informal sources (including friends and family), and 73% of respondents claimed to be aware of the risks of migrating to Europe. Given that a minority of respondents wished to return to Syria and there was a decrease in those wishing to return, any discussion or action on return by country officials may be contrary to displaced Syrians' aspirations and endanger their safety or well-being.

### Strengths and limitations of the study

The ADMIGOV project was conceived as a series of face-to-face interviews and ethnographic campaigns. The planned method was hampered by COVID-19 and related country regulations. We chose remote interviews as a result of talks among project partners and research into the best technique to reach Syrian-displaced populations in Lebanon. On the other hand, we leveraged the PI of the American University of Beirut team's previous experience and work to balance the impossibility of being on field and conducting ethnographic campaigns. The results demonstrated that this forced change in methodological tools materialised in the strength of our research. In fact, Syrians proved to be more relaxed over the phone and open to sharing their info, challenges and needs with us. Most of the interviews lasted longer than planned since the displaced needed to communicate with the outside environment to balance the feeling of loneliness that characterised the pandemic years. Moreover, since 2012 Syrians have been heavily on the hotspot of innumerable international and national organisations, which are repeatedly surveying and interviewing them. The physical distance imposed by the phone creates a relaxed condition between the participants that were also able to choose the time and date of the interview without interference with their daily life and housing environment. 

On the other hand, ethical concerns arise from using mobile phones to conduct surveys. Scholars have reported on the ethical challenges faced in their studies ( Ali *et al.,* 2017; Khalil *et al.,* 2021; Opdenakker, 2020). In our project, limiting the ethical issues required time and effort-consuming long preparation periods. It encompassed a reconsideration of the length of the questionnaires, clarity of the questions, translations of the interviews from English to Arabic and related translations of the responses from Arabic to English, and accurate training of the interviewers. Other limitations arose from the accrual of the acceptance of the consent form that was ratified orally after being accurately read in Arabic by the interviewer. The American University of Beirut's Institutional Review Board (IRB) played an important role throughout the preparatory period, helping us adjust and revise questions and consent forms. Another challenge materialised in recording the interviews while on the phone. Trials were conducted to assess the best solution.

### Implications of the project for the future

Our findings proved a series of gaps in the governance of Syrians displaced in Lebanon. The unclarity in the legal status recognition and related protection is paramount for reaching all displaced communities and ensuring aid and the recognition of their rights. At the end of our study, we deem it important to propose some recommendations according to our findings:

*Recommendation 1: on the subject of voluntary return*

Any discussion of return, as indicated by Fakhoury (2020) and Içduygu and Maissam (2020), is difficult since the conditions of safety and security are not assured. Furthermore, any repatriation effort directly harms displaced Syrians. Since 2017, the GSD has been returning Syrians to Lebanon, but there is no way to know whether it was voluntarily, and there is no means to ensure circumstances in Syria are safe. As a result, the essential advice is that authorities in hosting nations postpone any discussion or action related to the repatriation. A second proposal would be for the UN to establish a mechanism to monitor the safety of people wishing to return willingly.

*Recommendation 2: UN assistance with migration services and information*

Most Syrian displaced need to know their available alternatives on their migration journey. The United Nations, the International Organization for Migration, and other resettlement and humanitarian players should thus prioritise easy access and sharing of information on migration to ensure individuals are informed of the decision-making process. Given that most respondents' primary resource is a mobile phone, this may be accomplished via an Arabic audio or textual web page or the like.

*Recommendation 3: Financial Aid Adjusted for Currency Collapse*

As the value of the Lebanese Lira decreases, fewer goods and services may be purchased with it. As a result, it has become more difficult for displaced Syrians to achieve their basic nutritional requirements. Therefore, if donations arrive in Lebanon in USD or another foreign currency, they should be distributed proportionally to displaced Syrians, giving them greater freedom and buying power.

*Recommendation 4: Prioritize Health and Education Programs in Development Efforts*

Many respondents mentioned the lack of medical care for themselves or their children as a reason for wanting to leave. That made it harder for them to make choices. Many others also hoped to send their kids to school in another country. In conclusion, for Syrian displaced considering staying in Lebanon, access to quality education was the deciding factor. As a result, efforts to aid the displaced Syrian population should concentrate on these two areas of development.

*Recommendation 5: Maintaining Contact with Syrian Refugees*

Many Syrians involved in the study were overjoyed to provide their time and opinions, whether due to COVID-19, their displacement status, the difficult living circumstances in the country, or merely human nature. They were relieved to have an outlet for their thoughts and feelings. Therefore, humanitarian and development actors might contact displaced Syrian, searching for their conditions and daily lives, and provide mental health care to those in need.

## Data Availability

DANS: AdMiGov - Migration and Development - Lebanon. https://doi.org/10.17026/dans-x3h-t57x. ( Trovato, 2022b). The project contains the following underlying data: Clean Quantitative Data Lebanon.sav (Phone surveys results collected between February and July 2021 for 185 phone interviews with displaced Syrians residing in Bar Elias and Saadnayel in Zahle, and the Ouzai shelter in Saida) (restricted access). The data was collected as part of H2020 ADMIGOV project, subproject (WP6) that examined relationships between development interventions and the migration aspirations of refugees and other migrants. The data (phone surveys) was collected for a case study in Lebanon that focused on Syrian migration trends and development interventions. In total, between February and July 2021, 185 phone interviews with displaced Syrians residing in Bar Elias and Saadnayel in Zahle, and the Ouzai shelter in Saida were conducted. **Access rights:** The ADMIGOV consortium (WP leaders/Executive Board) decided that the project’s data will not be published for open access to protect confidentiality reasons. Therefore, the data will be accessible for verification purposes and re-use after 2028-01-30. Access rights/permission to use the data will be granted to registered DANS-EASY users upon a motivated request submitted to the data depositor (the PI of the AdMiGov) through the DANS-EASY repository. DANS: AdMiGov - Migration and Development - Lebanon. https://doi.org/10.17026/dans-x3h-t57x. ( Trovato, 2022b). This project contains the following extended data: Lebanon_Survey_Questionnaire.pdf. (Blank copy of the survey used in this study. (Restricted access). **Access rights:** The ADMIGOV consortium (WP leaders/Executive Board) decided that also the extended project’s data will not be published for open access. Therefore, the data will be accessible for verification purposes and re-use after 2028-01-30. Access rights/permission to use the data will be granted to registered DANS-EASY users upon a motivated request submitted to the data depositor (the PI of the AdMiGov) through the DANS-EASY repository.
