# Supplementary material for: Understanding Syrian migration in Lebanon: a methodological framework
Source: Open Res Eur. 2024 Jun 13;3:137. Originally published 2023 Sep 6. [Version 2] doi: 10.12688/openreseurope.16261.2 (PMC11214038; doi:10.12688/openreseurope.16261.2)
Supplement: Peer review history

# Reviewer report: Reviewer response for version 2

**DOI:** 10.21956/openreseurope.19284.r41444

**Published:** 2024-06-28

**Authors:** Jasmin Lilian Diab

**Affiliations:** 1 Institute for Migration Studies, Lebanese American University, Beirut, Beirut Governorate, Lebanon

Nothing to add.

If applicable, is the statistical analysis and its interpretation appropriate?

Not applicable

Is the study design appropriate and is the work technically sound?

Yes

Is the work clearly and accurately presented and does it engage with the current literature?

Yes

Are the conclusions drawn adequately supported by the results?

Partly

Are sufficient details of methods and analysis provided to allow replication by others?

Partly

Are all the source data and materials underlying the results available?

Yes

Reviewer Expertise:

Migration and Refugee Studies, Gender Studies and Conflict Studies with a focus on Lebanon and the broader Levant region including Syrian, Palestinian territories and Jordan.

I confirm that I have read this submission and believe that I have an appropriate level of expertise to confirm that it is of an acceptable scientific standard.

# Reviewer report: Reviewer response for version 2

**DOI:** 10.21956/openreseurope.19284.r41445

**Published:** 2024-06-20

**Authors:** Cenk Saracoglu

**Affiliations:** 1 Faculty of Communication, Ankara University, Ankara, Ankara, Turkey

The authors have effectively addressed the points raised by the reviewers, incorporating appropriate revisions that have significantly enhanced the manuscript's coherence and comprehensiveness. The revised version demonstrates substantial improvement over the initial submission. I recommend that the article be indexed in its current form.

If applicable, is the statistical analysis and its interpretation appropriate?

Yes

Is the study design appropriate and is the work technically sound?

Yes

Is the work clearly and accurately presented and does it engage with the current literature?

Partly

Are the conclusions drawn adequately supported by the results?

Partly

Are sufficient details of methods and analysis provided to allow replication by others?

Yes

Are all the source data and materials underlying the results available?

Yes

Reviewer Expertise:

Nationalism, migration, social theory, social inequalities

I confirm that I have read this submission and believe that I have an appropriate level of expertise to confirm that it is of an acceptable scientific standard.

# Reviewer report: Reviewer response for version 1

**DOI:** 10.21956/openreseurope.17555.r38551

**Published:** 2024-04-19

**Authors:** Jasmin Lilian Diab

**Affiliations:** 1 Institute for Migration Studies, Lebanese American University, Beirut, Beirut Governorate, Lebanon

**Summary:**

 The paper presents findings from a study conducted in Lebanon as part of the EU Horizon-funded project ADMIGOV, focusing on Syrian refugees' experiences in the country. The study employs a mixed-method approach, combining qualitative and quantitative data collection methods to shed light on the challenges faced by Syrian refugees living in informal settlements. While the paper offers valuable insights into the difficulties encountered by Syrian refugees in Lebanon and discusses the shortcomings of existing aid interventions, it falls short in exploring new intersections in the lived experiences of Syrian refugees and lacks an intersectional approach.

**Strengths:**

1. **Solid Methodology:** The paper demonstrates a robust methodological framework, incorporating a mixed-method approach that combines qualitative and quantitative data collection methods. The extensive fieldwork and immersion in the community contribute to the credibility of the study's findings.
2. **Relevance:** The topic of Syrian refugees' experiences in Lebanon is timely and relevant, given the ongoing crisis in Syria and the challenges faced by displaced populations in the region. The paper addresses an important issue and offers valuable insights for policymakers, practitioners, and researchers working in the field of forced migration and humanitarian responses.
3. **Practical Implications:** The findings of the study have practical implications for humanitarian actors and policymakers, highlighting the inadequacies of existing aid interventions and the need for more effective governance mechanisms to address the challenges faced by Syrian refugees in Lebanon.

**Weaknesses:**

1. **Lack of Exploration of New Intersections:** While the paper provides a comprehensive overview of the challenges faced by Syrian refugees in Lebanon, it does not explore any new intersections or phenomena in their lived experiences. The findings seem to reiterate existing knowledge without offering any novel insights into the complexities of Syrian refugees' experiences in Lebanon.
2. **Absence of Intersectional Approach:** The paper lacks an intersectional approach, as the findings are presented in a rather generalized manner, and the population of Syrian refugees is homogenized. There is a missed opportunity to explore the intersecting factors that shape the experiences of different subgroups within the Syrian refugee population, such as gender, age, socioeconomic status, and educational background.
3. **Qualitative Findings Need Further Development:** While the qualitative findings provide valuable insights into the challenges faced by Syrian refugees in Lebanon, they could be further developed to offer more nuanced and 'new' approaches to the research question. The paper primarily focuses on highlighting challenges matter-of-factly without delving into the underlying dynamics and complexities of the refugee experience.

**Recommendations:**

1. **Develop New Intersections:** The authors should endeavor to explore new intersections in the lived experiences of Syrian refugees in Lebanon, moving beyond existing knowledge to uncover novel insights into the complexities of displacement and forced migration. This could involve considering factors such as gender, age, socioeconomic status, and educational background and examining how these intersecting identities shape refugees' experiences and outcomes.
2. **Adopt an Intersectional Approach:** The paper would benefit from adopting an intersectional approach to analyze the findings, recognizing the diversity within the Syrian refugee population and exploring how intersecting factors influence their experiences and vulnerabilities. This would provide a more nuanced understanding of the challenges faced by different subgroups within the refugee population.
3. **Use Qualitative Findings to Develop Nuanced Approaches:** The authors should utilize the qualitative findings to develop more nuanced and 'new' approaches to the research question, moving beyond merely highlighting challenges to uncovering the underlying dynamics and complexities of the refugee experience. This could involve conducting further qualitative analysis to explore themes and patterns in greater depth and developing recommendations based on these insights.

Overall, while the paper presents a solid analysis of the challenges faced by Syrian refugees in Lebanon, there is room for improvement in terms of exploring new intersections, adopting an intersectional approach, and using qualitative findings to develop more nuanced approaches to the research question. With these revisions, the paper has the potential to make a significant contribution to the field of forced migration and humanitarian responses.

If applicable, is the statistical analysis and its interpretation appropriate?

Not applicable

Is the study design appropriate and is the work technically sound?

Yes

Is the work clearly and accurately presented and does it engage with the current literature?

Yes

Are the conclusions drawn adequately supported by the results?

Partly

Are sufficient details of methods and analysis provided to allow replication by others?

Partly

Are all the source data and materials underlying the results available?

Yes

Reviewer Expertise:

Migration and Refugee Studies, Gender Studies and Conflict Studies with a focus on Lebanon and the broader Levant region including Syrian, Palestinian territories and Jordan.

I confirm that I have read this submission and believe that I have an appropriate level of expertise to confirm that it is of an acceptable scientific standard, however I have significant reservations, as outlined above.

# Response

**Published:** 2024-05-22

**Authors:** Maria Gabriella Trovato

**Affiliations:** Landscape Design and Ecosystem Management, American University of Beirut, Beirut, Beirut Governorate, Lebanon

Dear, Thank you for allowing us to adjust our paper. Below is the list of issues you asked us to address and our responses. ***Weaknesses:***

1. ***Lack of Exploration of New Intersections:*** *While the paper provides a comprehensive overview of the challenges faced by Syrian refugees in Lebanon, it does not explore any new intersections or phenomena in their lived experiences. The findings seem to reiterate existing knowledge without offering any novel insights into the complexities of Syrian refugees' experiences in Lebanon.*
2. ***Absence of Intersectional Approach:*** *The paper lacks an intersectional approach, as the findings are presented in a rather generalized manner, and the population of Syrian refugees is homogenized. There is a missed opportunity to explore the intersecting factors that shape the experiences of different subgroups within the Syrian refugee population, such as gender, age, socioeconomic status, and educational background.*
3. ***Qualitative Findings Need Further Development:*** *While the qualitative findings provide valuable insights into the challenges faced by Syrian refugees in Lebanon, they could be further developed to offer more nuanced and 'new' approaches to the research question. The paper primarily focuses on highlighting challenges matter-of-factly without delving into the underlying dynamics and complexities of the refugee experience.*

Your list of weaknesses made us reconsider the way we presented our analysis. So, we have reworked the discussion and conclusion of our paper, adding on the dynamics of the dual power relation between political inability and Syrian social agency in the shadow of the formal structure of the city and rural areas. We highlighted the presence of patterns to navigate the difficulties in daily life and the role of the spatial location of the displaced in their capacity to cope with the struggles and their aspirations for the future. ***Recommendations:***

1. ***Develop New Intersections:*** *The authors should endeavor to explore new intersections in the lived experiences of Syrian refugees in Lebanon, moving beyond existing knowledge to uncover novel insights into the complexities of displacement and forced migration. This could involve considering factors such as gender, age, socioeconomic status, and educational background and examining how these intersecting identities shape refugees' experiences and outcomes. *

This study didn’t address the individuality of the refugee experience. Instead, we assessed how the Lebanese political decision of denying legal status to refugees, determining the spread of Syrians across the country, has resulted in unequal treatment and increased marginalisation and vulnerability of the displaced based on their localisation. The study revealed that communities located in ITS in rural areas benefitted more from UN assistance than Syrians living in the city’s apartments. Some info on gender has been added in the section on aspiration and the reworked discussion and conclusion.  

1. ***Adopt an Intersectional Approach:*** *The paper would benefit from adopting an intersectional approach to analyze the findings, recognizing the diversity within the Syrian refugee population and exploring how intersecting factors influence their experiences and vulnerabilities. This would provide a more nuanced understanding of the challenges faced by different subgroups within the refugee population.*

While we recognise the importance of the reviewer’s recommendation, we want to emphasise that our study used a case study approach aimed at assessing and highlighting the importance of the type of settlings and their location on the well-being, disparities in treatment, marginalisation and vulnerability of Syrians in Lebanon. Therefore, we didn’t look at individuality but at the community and geopolitical differences and how they relate to the governance of the migratory phenomena and development aid.

1. ***Use Qualitative Findings to Develop Nuanced Approaches:*** *The authors should utilize the qualitative findings to develop more nuanced and 'new' approaches to the research question, moving beyond merely highlighting challenges to uncovering the underlying dynamics and complexities of the refugee experience. This could involve conducting further qualitative analysis to explore themes and patterns in greater depth and developing recommendations based on these insights* *. *

Thank you for your insightful comment. To answer your recommendation, we identified themes and patterns in the discussion and conclusion and developed new recommendations based on these insights.

# Reviewer report: Reviewer response for version 1

**DOI:** 10.21956/openreseurope.17555.r34971

**Published:** 2023-09-27

**Authors:** Cenk Saracoglu

**Affiliations:** 1 Faculty of Communication, Ankara University, Ankara, Ankara, Turkey

This research paper endeavors to present an all-encompassing examination of the plight of "displaced Syrians" within the borders of Lebanon. This undertaking is accomplished through an extensive fieldwork initiative, conducted in both the Bekaa Valley and the city of Saida. The fieldwork is an amalgamation of various qualitative and quantitative research methodologies, including in-depth interviews, surveys, and ethnographic observations. The authors aptly argue that an array of stressors, notably the relentless surge in inflation due to an economic crisis, the omnipresent specter of the Covid-19 pandemic, and the devastating impact of the Beirut port disasters on Lebanese society, have grievously exacerbated the already precarious living conditions of the Syrian population. This confluence of challenges has not only magnified the Syrians' predicaments but also convoluted the issues they confront.

 In the absence of a formal domestic framework that could potentially offer protection and ameliorate the vulnerability of displaced Syrians, the reliance on informal community support and existing formal assistance mechanisms proves insufficient to shield them from perilous circumstances, abject poverty, and marginalization. Regrettably, these hardships have driven a substantial majority of Syrians to contemplate leaving the host country, albeit with limited prospects of success.

 In light of these pressing issues, the authors judiciously proffer a set of policy recommendations. These include advocating for the postponement of forced repatriation, enhancing the capacity of international humanitarian institutions to facilitate displaced individuals' access to vital information, adjusting the scope and magnitude of financial aid packages in accordance with the precipitous currency devaluation, and focusing concerted efforts on the improvement of health and education programs.

 This research paper is undeniably a well-crafted, logically structured, and cohesive exploration that vividly portrays the current predicament of Syrian refugees amid the multiple crises afflicting Lebanese society. It also proffers a concrete set of policy prescriptions. However, the wealth of research material upon which this paper is founded could have been more effectively leveraged to delve into the following areas:

1. While acknowledging the existence of prior research on displaced Syrians in Lebanon, such as the works of Fakhoury, Janmyr, and Romola, the authors could have further elaborated on how their current findings either corroborate or challenge the conclusions of these scholars. This comparative analysis would have enriched the discourse.
2. The authors might have delved deeper into the data derived from their quantitative and qualitative interviews to expound upon the stark disparities in aspirations and priorities between displaced Syrians in Bekaa and Saida. By shedding light on the underlying determinants of these differences, the paper could have offered insights into how the socio-economic circumstances and living conditions of refugees collectively shape their hopes, expectations, and, consequently, their decisions regarding relocation.
3. While acknowledging that the influx of Syrians has altered the ethnic and sectarian composition of Lebanese cities and towns, exacerbating social tensions and fostering racial prejudices, the research paper, in its current form, falls short in adequately addressing how displaced Syrians experience these tensions. Additionally, there is room for a more comprehensive exploration of how the degree and nature of negative attitudes within the local population have evolved in tandem with Lebanon's multifaceted crises and how these experiences influence the dire conditions faced by the displaced population. Given that the authors mention their interviews aimed to gauge the strength of Syrians' ties to local communities, readers would benefit from an analysis of how intercommunal relations between the local Lebanese community and displaced Syrians evolve over time.
4. Numerous studies conducted in Turkey on the conditions of Syrian asylum seekers document similar challenges to those experienced by Syrians in Lebanon. Incorporating insights from this extensive body of research in Turkey, juxtaposed with the Lebanese context, could provide valuable comparative analysis, highlighting the ways in which Lebanon's unique political, legal, and social conditions exert a distinct influence on the Syrian population within its borders.
5. In light of the prevailing structural constraints, notably the absence of a robust domestic legal framework designed to safeguard the rights and well-being of Syrians residing in Lebanon, it becomes increasingly questionable whether the ostensibly modest policy recommendations put forth by the researchers can yield any discernible and positive impact on the dire circumstances faced by the Syrian population. An astute examination of the limitations inherent in these short-term and relatively small-scale policy proposals could have imparted a greater degree of nuance and sophistication to the authors' discussion. By addressing this crucial concern, the paper could have embraced a more comprehensive perspective, acknowledging the formidable hurdles posed by the overarching legal and institutional deficiencies within Lebanon. In doing so, it would have offered readers a more well-rounded understanding of the complexities inherent in effecting change in the lives of displaced Syrians within such a challenging and constrained environment.

If applicable, is the statistical analysis and its interpretation appropriate?

Yes

Is the study design appropriate and is the work technically sound?

Yes

Is the work clearly and accurately presented and does it engage with the current literature?

Partly

Are the conclusions drawn adequately supported by the results?

Partly

Are sufficient details of methods and analysis provided to allow replication by others?

Yes

Are all the source data and materials underlying the results available?

Yes

Reviewer Expertise:

Nationalism, migration, social theory, social inequalities

I confirm that I have read this submission and believe that I have an appropriate level of expertise to confirm that it is of an acceptable scientific standard, however I have significant reservations, as outlined above.

# Response

**Published:** 2024-05-22

**Authors:** Maria Gabriella Trovato

**Affiliations:** Landscape Design and Ecosystem Management, American University of Beirut, Beirut, Beirut Governorate, Lebanon

Dear, Thank you for allowing us to adjust our paper. Below is the list of issues you asked us to address and our responses.

1. *While acknowledging the existence of prior research on displaced Syrians in Lebanon, such as the works of Fakhoury, Janmyr, and Romola, the authors could have further elaborated on how their current findings either corroborate or challenge the conclusions of these scholars. This comparative analysis would have enriched the discourse. *

 Throughout the paper, our data analysis has been compared with the results of other scholars' work, even though there is not a dedicated section of the paper on this comparative work.

1. *The authors might have delved deeper into the data derived from their quantitative and qualitative interviews to expound upon the stark disparities in aspirations and priorities between displaced Syrians in Bekaa and Saida. By shedding light on the underlying determinants of these differences, the paper could have offered insights into how the socio-economic circumstances and living conditions of refugees collectively shape their hopes, expectations, and, consequently, their decisions regarding relocation. *

We answered this suggestion by reworking the section of the paper on the respondents' aspirations and priorities. Moreover, we added a paragraph in the discussion and conclusion. (Please see the part … Location appears to also affect the aspirations of displaced Syrians, with those living in the city dreaming of leaving and going abroad, while Syrians from Bekaa have the goals of improving their standard of living and meeting their basic needs.)

1. *While acknowledging that the influx of Syrians has altered the ethnic and sectarian composition of Lebanese cities and towns, exacerbating social tensions and fostering racial prejudices, the research paper, in its current form, falls short in adequately addressing how displaced Syrians experience these tensions. Additionally, there is room for a more comprehensive exploration of how the degree and nature of negative attitudes within the local population have evolved in tandem with Lebanon's multifaceted crises and how these experiences influence the dire conditions faced by the displaced population. Given that the authors mention their interviews aimed to gauge the strength of Syrians' ties to local communities, readers would benefit from an analysis of how intercommunal relations between the local Lebanese community and displaced Syrians evolve over time*

To answer this remark, we added a part at the beginning of the **discussion and conclusion **and reworked the **introduction **to clarify this point from the beginning of the article.

1. *Numerous studies conducted in Turkey on the conditions of Syrian asylum seekers document similar challenges to those experienced by Syrians in Lebanon. Incorporating insights from this extensive body of research in Turkey, juxtaposed with the Lebanese context, could provide valuable comparative analysis, highlighting the ways in which Lebanon's unique political, legal, and social conditions exert a distinct influence on the Syrian population within its borders. *

We are aware of the importance of comparing migrants' conditions in different countries. However, our paper's scope was different. Nonetheless, we are in touch with our Horizon research partner in Turkey, and we have discussed writing a paper comparing the data from Lebanon and Turkey.


* *

1. *In light of the prevailing structural constraints, notably the absence of a robust domestic legal framework designed to safeguard the rights and well-being of Syrians residing in Lebanon, it becomes increasingly questionable whether the ostensibly modest policy recommendations put forth by the researchers can yield any discernible and positive impact on the dire circumstances faced by the Syrian population. An astute examination of the limitations inherent in these short-term and relatively small-scale policy proposals could have imparted a greater degree of nuance and sophistication to the authors' discussion. By addressing this crucial concern, the paper could have embraced a more comprehensive perspective, acknowledging the formidable hurdles posed by the overarching legal and institutional deficiencies within Lebanon. In doing* so, *it would have offered readers a more well-rounded understanding of the complexities inherent in effecting change in the lives of displaced Syrians within such a challenging and constrained environment. *

We are aware of the modesty of the proposed policy recommendations compared to the complexity of the governance of the phenomena. Therefore, we have developed new recommendations based on the reworked discussion and conclusions.
